# Effects of bystander programs on the prevention of sexual assault among adolescents and college students: A systematic review

**DOI:** 10.4073/csr.2019.1

**Published:** 2019-07-18

**Authors:** Heather Hensman Kettrey, Robert A. Marx, Emily E. Tanner‐Smith

**Affiliations:** ^1^ Department of Sociology, Anthropology, and Criminal Justice Clemson University Clemson South Carolina; ^2^ Department of Child and Adolescent Development, Lurie College San Jose State University San Jose California; ^3^ Department of Counseling Psychology and Human Services University of Oregon Eugene Oregon

## 1 PLAIN LANGUAGE SUMMARY

### 1.1 Bystander programs increase bystander intervention but no effect on perpetrating sexual assault

Bystander sexual assault prevention programs have beneficial effects on bystander intervention but there is no evidence of effects on sexual assault perpetration. Effects on knowledge and attitudes are inconsistent across outcomes.

### 1.2 What is this review about?

Sexual assault is a significant problem among adolescents and college students across the world. One promising strategy for preventing these assaults is the implementation of bystander sexual assault prevention programs, which encourage young people to intervene when witnessing incidents or warning signs of sexual assault. This review examines the effects bystander programs have on knowledge and attitudes concerning sexual assault and bystander behavior, bystander intervention when witnessing sexual assault or its warning signs, and participants’ rates of perpetration of sexual assault.

**What is the aim of this review?**

This Campbell systematic review examines the effects of bystander programs on knowledge and attitudes concerning sexual assault and bystander intervention, bystander intervention when witnessing sexual assault or its warning signs, and the perpetration of sexual assault. The review summarizes evidence from 27 high‐quality studies, including 21 randomized controlled trials.

## 2 WHAT ARE THE MAIN FINDINGS OF THIS REVIEW?

### 2.1 What studies are included?

This review includes studies that evaluate the effects of bystander programs for young people on (a) knowledge and attitudes concerning sexual assault and bystander intervention, (b) bystander intervention behavior when witnessing sexual assault or its warning signs, and (c) perpetration of sexual assault. Twenty‐seven studies met the inclusion criteria. These included studies span the period from 1997 to 2017 and were primarily conducted in the USA (one study was conducted in Canada and one in India). Twenty‐one studies were randomized controlled trials and six were high quality quasi‐experimental studies.

### 2.2 Do bystander programs have an effect on knowledge/attitudes, on bystander intervention, or on sexual assault perpetration?

Bystander programs have an effect on knowledge and attitudes for some outcomes. The most pronounced beneficial effects are on rape myth acceptance and bystander efficacy outcomes. There are also delayed effects (i.e., 1 to 4 months after the intervention) on taking responsibility for intervening/acting, knowing strategies for intervening, and intentions to intervene outcomes. There is little or no evidence of effects on gender attitudes, victim empathy, date rape attitudes, and on noticing sexual assault outcomes.

Bystander programs have a beneficial effect on bystander intervention. There is no evidence that bystander programs have an effect on participants’ rates of sexual assault perpetration.

## 3 WHAT DO THE FINDINGS OF THIS REVIEW MEAN?

The United States 2013 Campus Sexual Violence Elimination (SaVE) Act requires postsecondary educational institutions participating in Title IX financial aid programs to provide incoming college students with sexual violence prevention programming that includes a component on bystander intervention.

Bystander programs have a significant effect on bystander intervention. But there is no evidence that these programs have an effect on rates of sexual assault perpetration. This suggests that bystander programs may be appropriate for targeting the behavior of potential bystanders but may not be appropriate for targeting the behavior of potential perpetrators.

Beneficial effects of bystander programs on bystander intervention were diminished by 6 months post‐intervention. Thus, booster sessions may be needed to yield any sustained effects.

There are still important questions worth further exploration. Namely, more research is needed to investigate the underlying causal mechanisms of program effects on bystander behavior (e.g., to model relationships between specific knowledge/attitude effects and bystander intervention effects), and to identify the most effective types of bystander programs (e.g., using randomized controlled trials to compare the effects of two alternate program models). Additionally, more research is needed in contexts outside of the USA so that researchers can better understand the role of bystander programs across the world.

## 4 HOW UP‐TO‐DATE IS THIS REVIEW?

The review authors searched for studies up to June 2017. This Campbell systematic review was submitted in October 2017, revised in October 2018, and published January 2019.

## 5 EXECUTIVE SUMMARY/ABSTRACT

### 5.1 Background

#### 5.1.1 Sexual assault among adolescents and college students

Sexual assault is a significant problem among adolescents and college students in the United States and globally. Findings from the Campus Sexual Assault study estimated that 15.9% of college women had experienced attempted or completed sexual assault (i.e., unwanted sexual contact that could include sexual touching, oral sex, intercourse, anal sex, or penetration with a finger or object) prior to entering college and 19% had experienced attempted or completed sexual assault since entering college (Krebs, Lindquist, Warner, Fisher, & Martin, 2009). Similar rates have been reported in Australia (Australian Human Rights Commission, 2017), Chile (Lehrer, Lehrer, & Koss, 2013), China (Su, Hao, Huang, Xiao, & Tao, 2011), Finland (Bjorklund, Hakkanen‐Nyholm, Huttunen, & Kunttu, 2010), Poland (Tomaszewska & Krahe, 2018), Rwanda (Van Decraen, Michielsen, Herbots, Van Rossem, & Temmerman, 2012), Spain (Vázquez, Torres, & Otero, 2012), and in a global survey of countries in Africa, Asia, and the Americas (Pengpid & Peltzer, 2016).

#### 5.1.2 The bystander approach

One promising strategy for preventing sexual assault among adolescents and young adults is the implementation of bystander programs, which encourage young people to intervene when witnessing incidents or warning signs of sexual assault. Bystander programs seek to sensitize young people to warning signs of sexual assault, create attitudinal changes that foster bystander responsibility for intervening (e.g., creating empathy for victims), and build requisite skills and knowledge of tactics for taking action (Banyard, 2011; Banyard, Plante, & Moynihan, 2004; Burn, 2009; McMahon & Banyard, 2012). Many of these programs are implemented with large groups of adolescents or college students in the format of a single training/education session (e.g., as part of college orientation). However, some programs use broader implementation strategies, such as advertising campaigns that post signs across college campuses to encourage students to act when witnessing signs of violence.

By treating young people as potential allies in preventing sexual assault, bystander programs have the capacity to be less threatening than traditional sexual assault prevention programs, which tend to address young people as either potential perpetrators or victims of sexual violence (Burn, 2009; Messner, 2015; [Jackson] Katz, 1995). Instead of placing emphasis on how young people may modify their individual behavior to either respect the sexual boundaries of others or reduce their personal risk for being sexually assaulted, bystander programs aim to foster prerequisite knowledge and skills for intervening on behalf of potential victims. Thus, by treating young people as part of the solution to sexual assault, rather than part of the problem, bystander programs may limit the risk of defensiveness or backlash among participants (e.g., decreased empathy for victims, increased rape myth acceptance; Banyard et al., 2004; Katz, 1995).

### 5.2 Objectives

The overall objective of this systematic review and meta‐analysis was to examine what effects bystander programs have on preventing sexual assault among adolescents and college students. More specifically, this review addressed three objectives.

1. The first objective was to assess the overall effects (including adverse effects), and the variability of the effects, of bystander programs on adolescents’ and college students’ attitudes and behaviors regarding sexual assault.
2. The second objective was to explore the comparative effectiveness of bystander programs for different profiles of participants (e.g., mean age of the sample, education level of the sample, proportion of males/females in the sample, proportion of fraternity/sorority members in the sample, proportion of athletic team members in the sample).
3. The third objective was to explore the comparative effectiveness of different bystander programs in terms of gendered content and approach (e.g., conceptualizing sexual assault as a gendered or gender‐neutral problem, mixed‐ or single‐sex group implementation).

### 5.3 Search methods

Candidate studies were identified through searches of electronic databases, relevant academic journals, and gray literature sources. Gray literature searches included contacting leading authors and experts on bystander programs to identify any current/ongoing research that might be eligible for the review, screening bibliographies of eligible studies and relevant reviews to identify additional candidate studies, and conducting forward citation searches (searches for reports citing eligible studies) using the website Google Scholar.

### 5.4 Selection criteria

To be included in the review studies had to meet eligibility criteria in the following domains: types of study, types of participants, types of interventions, types of outcome measures, duration of follow‐up, and types of settings.

#### 5.4.1 Types of studies

To be eligible for inclusion in the review, studies must have used an experimental or controlled quasi‐experimental research design to compare an intervention group (i.e., students assigned to a bystander program) with a comparison group (i.e., students not assigned to a bystander program).

#### 5.4.2 Types of participants

The review focused on studies that examined outcomes of bystander programs that targeted sexual assault and were implemented with adolescents and/or college students in educational settings. Eligible participants included adolescents enrolled in grades 7 through 12 and college students enrolled in any type of undergraduate postsecondary educational institution. The mean age of samples could be no less than age 12 and no greater than age 25.

#### 5.4.3 Types of interventions

Eligible intervention programs were those that approached participants as allies in preventing and/or alleviating sexual assault among adolescents and/or college students. Some part of the program had to focus on ways that cultivate willingness for a person to respond to others who are at risk for sexual assault. All delivery formats were eligible for inclusion (e.g., in‐person training sessions, video programs, web‐based training, and advertising/poster campaigns). There were no intervention duration criteria for inclusion.

Eligible comparison groups must have received no intervention services targeting bystander attitudes/behavior or sexual assault.

#### 5.4.4 Types of outcome measures

We included studies that measured the effects of bystander programs on at least one of the following primary outcome domains:

1. General attitudes toward sexual assault and victims (e.g., victim empathy, rape myth acceptance).
2. Prerequisite skills and knowledge for bystander intervention as defined by Burn (2009) (e.g., noticing sexual assault or its warning signs, identifying a situation as appropriate for intervention, taking responsibility for acting/intervening, and knowing strategies for helping/intervening).
3. Self‐efficacy with regard to bystander intervention (e.g., respondents’ confidence in their ability to intervene).
4. Intentions to intervene when witnessing instances or warning signs of sexual assault.
5. Actual intervention behavior when witnessing instances or warning signs of sexual assault.
6. Perpetration of sexual assault (i.e., participants’ rates of perpetration).

#### 5.4.5 Duration of follow‐up

Studies reporting follow‐ups of any duration were eligible for inclusion. When studies reported outcomes at more than one follow‐up wave, each wave was coded and identified by its reported duration. Follow‐ups of similar durations were analyzed together.

#### 5.4.6 Types of settings

The review focused on studies that examined outcomes of bystander programs that targeted sexual assault and were implemented with adolescents and/or college students in educational settings. Eligible educational settings included secondary schools (i.e., grades 7–12) and colleges or universities. There were no geographic limitations on inclusion criteria. Research conducted in any country was eligible.

### 5.5 Data collection and analysis

#### 5.5.1 Selection of studies

Once candidate studies were identified, two reviewers independently screened each study title and abstract for eligibility; disagreements between reviewers were resolved by discussion and consensus. Potentially eligible studies were then retrieved in full text, and these full texts were reviewed for eligibility, again using two independent reviewers.

#### 5.5.2 Data extraction and management

Two reviewers independently double‐coded all included studies, using a piloted codebook. Coding disagreements were resolved via discussion and consensus. The primary categories for coding were as follows: participant demographics and characteristics (e.g., age, gender, education level, race/ethnicity, athletic team membership, fraternity/sorority membership); intervention setting (e.g., state, country, secondary or postsecondary institution, mixed‐ or single‐sex group); study characteristics (e.g., attrition, duration of follow‐up, study design, participant dose, sample *N*); outcome construct (e.g., type, description of measure); and outcome results (e.g., timing at measurement, baseline, and follow‐up means and standard deviations or proportions).

#### 5.5.3 Measures of treatment effect

During the coding process, relevant summary statistics (e.g., means and standard deviations, proportions, observed sample sizes) were extracted from research reports to calculate effect sizes. Effect sizes were reported as standardized mean differences (SMD), adjusted for small sample size (Hedges’ *g*). Positive effect size values (i.e., greater than 0) indicate a beneficial outcome for the bystander intervention group.

#### 5.5.4 Data synthesis

Intervention effects for each outcome construct were synthesized via meta‐analyses using random‐effects inverse variance weights. All statistical analyses were conducted with the metafor package in *R*. Synthesis results are displayed using forest plots. Mean effect sizes are reported with their 95% confidence intervals.

### 5.6 Results

#### 5.6.1 Objective 1: Effects on knowledge, attitudes, and behavior

##### Knowledge/Attitudes

Effects for knowledge and attitude outcomes varied widely across constructs. The most pronounced beneficial effect in this domain was on rape myth acceptance. The effect for this outcome was immediate and sustained across all reported follow‐up waves (i.e., from immediate posttest to 6‐ to 7‐month post‐intervention). Intervention effects on bystander efficacy were also fairly pronounced, with an effect observed at both immediate posttest and 1‐ to 4‐month post‐intervention. A significant effect was not observed for this outcome 6 months post‐intervention; however, this should be interpreted with caution, as only one study reported bystander efficacy effects at this follow‐up period.

Effects on other knowledge and attitude outcomes were either delayed or unobserved. Intervention effects on taking responsibility for intervening or acting, knowing strategies for intervening, and intentions to intervene were nonsignificant at immediate posttest, but significant and beneficial by 1‐ to 4‐month post‐intervention. We found limited or no evidence of significant intervention effects on gender attitudes, victim empathy, date rape attitudes, and noticing sexual assault.

##### Behavior

The results indicated that bystander programs have a beneficial effect on bystander intervention behavior. However, this effect, which was observed at 1‐ to 4‐month post‐intervention, was not statistically significant at 6 months post‐intervention. Bystander programs did not have a significant effect on sexual assault perpetration.

#### 5.6.2 Objective 2: Effects for different participant profiles

We had planned to conduct moderator analyses to assess a wide range of participant characteristics as potential effect size moderators. However, our review only yielded a sufficient number of studies (*n* ≥ 10) to conduct moderator analyses for the bystander intervention outcome domain. The results indicated that mean age, education level, and proportion of males/female were not statistically significant predictors of the magnitude of intervention effects.

#### 5.6.3 Objective 3: Effects based on gendered content/implementation

We conducted moderator analyses to assess any differential effects of bystander programs on measured outcomes based on (a) the gender of perpetrators and victims in specific bystander programs and (b) whether programs were implemented in mixed‐ or single‐sex settings. Our review only produced a sufficient number of studies (*n* ≥ 10) to conduct such moderator analyses for the bystander intervention outcome domain. We found that neither of these measures was a significant predictor of the effectiveness of bystander programs on bystander intervention.

### 5.7 Authors’ conclusions

#### 5.7.1 Implications for practice and policy

The overwhelming majority of eligible studies assessing the effects of bystander programs were conducted in the United States. This is not necessarily surprising considering that the United States has implemented public policy that encourages the implementation of such programs on college campuses. The United States 2013 Campus SaVE Act requires postsecondary educational institutions participating in Title IX financial aid programs to provide incoming college students with primary prevention and awareness programs addressing sexual violence. The Campus SaVE Act mandates that these programs include a component on bystander intervention. Currently, there is no comparable legislation regarding sexual assault among adolescents (e.g., mandating bystander programs in secondary schools).

Findings from this review indicate that bystander programs have beneficial effects on bystander intervention behavior. This review therefore provides important evidence of the effectiveness of mandated programs on college campuses. Additionally, results from the moderator analyses indicated that the effects on bystander intervention are similar for adolescents and college students, which suggests that early implementation of bystander programs (i.e., in secondary schools with adolescents) may be warranted.

Importantly, although we found that bystander programs had a significant effect on bystander intervention behavior, there was no evidence that these programs had an effect on participants’ sexual assault perpetration. Although most bystander sexual assault prevention programs aim to shift attitudes in the hopes of preventing sexual assault perpetration, this review provided no evidence that these programs decrease participants’ perpetration rates. This suggests that bystander programs may be appropriate for targeting bystander behavior but may not be appropriate for targeting the behavior of potential perpetrators. Additionally, effects of bystander programs on bystander intervention diminished 6‐month post‐intervention. Thus, booster sessions may be needed to yield any sustained intervention effects.

#### 5.7.2 Implications for research

Findings from this review suggest there is a fairly strong body of research assessing the effects of bystander programs on attitudes and behaviors. However, there are important questions worth further exploration.

First, according to one prominent logic model, bystander programs promote bystander intervention by fostering prerequisite knowledge and attitudes (Burn, 2009). Our meta‐analysis provides inconsistent evidence of the effects of bystander programs on knowledge and attitudes, but promising evidence of short‐term effects on bystander intervention behavior. These results cast uncertainty on the proposed relationship between knowledge/attitudes and bystander behavior. However, our methods do not permit any formal evaluation of this relationship. The field's understanding of the causal mechanisms of program effects on bystander behavior would benefit from further analysis (e.g., path analysis mapping relationships between specific knowledge/attitude effects and bystander intervention).

Second, bystander programs exhibit a great deal of content variability, most notably in framing sexual assault as a gendered or gender‐neutral problem. That is, bystander programs tend to adopt one of two main approaches to addressing sexual assault: (a) they present sexual assault as a gendered problem (overwhelmingly affecting women) or (b) they present sexual assault as a gender‐neutral problem (affecting women and men alike). Differential effects of these two types of programs remain largely unexamined. Our analysis indicated that (a) the sex of victims/perpetrators presented in interventions (i.e., portrayed in programs as gender neutral or male perpetrator and female victim) and (b) whether programs were implemented in mixed‐ or single‐sex settings were not significant predictors of program effects on bystander intervention. However, these findings are limited to a single outcome and they should be considered preliminary, as they are based on a small sample (*n* = 11). The field's understanding of the differential effects of gendered versus gender neutral programs would benefit from the design and implementation of high‐quality primary studies that make direct comparisons between these two types of programs (e.g., randomized controlled trials [RCTs] comparing the effects of two active treatment arms that differ in their gendered approach).

Finally, as previously noted, all but two eligible studies were conducted in the United States. Thus, high‐quality studies conducted outside of the United States are needed to provide a global perspective on the efficacy of bystander programs.

## 6 BACKGROUND

### 6.1 The problem, condition, or issue

#### 6.1.1 Sexual assault among adolescents and college students

Sexual assault is a significant problem among adolescents and college students in the United States and globally. Findings from the Campus Sexual Assault study estimated that 15.9% of American college women had experienced attempted or completed sexual assault (i.e., unwanted sexual contact that could include sexual touching, oral sex, intercourse, anal sex, or penetration with a finger or object) prior to entering college and 19% had experienced attempted or completed sexual assault since entering college (Krebs et al., 2009).

Similar rates have been reported in Australia (Australian Human Rights Commission, 2017), Chile (Lehrer et al., 2013), China (Su et al., 2011), Finland (Bjorklund et al., 2010), Poland (Tomaszewska & Krahe, 2018), Rwanda (Van Decraen et al., 2012), Spain (Vázquez et al., 2012), and in a global survey of countries in Africa, Asia, and the Americas (Pengpid & Peltzer, 2016).

These rates are problematic, as sexual assault in adolescence and/or young adulthood is associated with numerous adverse outcomes, including risk of repeated victimization, depressive symptomology, heavy drinking, and suicidal ideation (Exner‐ Cortens, Eckenrode, & Rothman, 2013; Cui, Ueno, Gordon, & Fincham, 2013; Halpern, Spriggs, Martin, & Kupper, 2009). Importantly, there is evidence that indicates that experiences of sexual assault during these two life phases are related as victimization and perpetration during adolescence, respectively, associated with increased risk of victimization and perpetration during young adulthood (Cui et al., 2013). Thus, early prevention efforts are of paramount importance.

Reviews of research on the effectiveness of programs designed to prevent sexual assault among adolescents and college students have noted both a dearth of high‐quality studies, such as RCTs and minimal evidence that these prevention programs have meaningful effects on young people's behavior (De Koker, Mathews, Zuch, Bastien, & Mason‐Jones, 2014; DeGue et al., 2014). Concerning the latter point, evaluations of such programs tend to measure attitudinal outcomes (e.g., rape supportive attitudes, rape myth acceptance) more frequently than behavioral outcomes (e.g., perpetration or victimization; Anderson & Whiston, 2005; Cornelius & Resseguie, 2007; DeGue et al., 2014). Additionally, findings from a meta‐analysis of studies assessing outcomes of college sexual assault prevention programs suggested that effects are larger for attitudinal outcomes than for the actual incidence of sexual assault (Anderson & Whiston, 2005).

### 6.2 The intervention

#### 6.2.1 The bystander approach

Given this paucity of evidence regarding behavior change, it is imperative to identify effective strategies for preventing sexual assault among adolescents and young adults. One promising strategy is the implementation of bystander programs, which encourage young people to intervene when witnessing incidents or warning signs of sexual assault (e.g., controlling behavior, such as intervening with a would‐be perpetrator leading an intoxicated person into an isolated area). The strength of the bystander model lies in its emphasis on the role of peers in the prevention of violence. Peers are a salient influence on young people's intimate relationships (Adelman & Kil, 2007; Giordano, 2003). In some respects, this influence can be detrimental, as having friends involved in violent intimate relationships (i.e., characterized by sexual or physical violence) is a risk factor for becoming both a perpetrator and victim of violence (Arriaga & Foshee, 2004; Foshee, Benefield, Ennett, Bauman, & Suchindran, 2004; Foshee, Linder, MacDougall, & Bangdiwala, 2001; Foshee, Reyes, & Ennett, 2010; McCauley et al., 2013). However, peers can also have a positive impact on intimate relationships.

Young victims and perpetrators of violence are often reluctant to divulge their experience or to seek help (especially from adults), but when they do seek help they often seek it from their peers (Ashley & Foshee, 2005; Black, Tolman, Callahan, Saunders, & Weisz, 2008; Molidor & Tolman, 1998; Weisz, Tolman, Callahan, Saunders, & Black, 2007). Victims may trust their peers to provide a valuable source of support after an assault has occurred, and as such, peers have the potential to play a pivotal role in the prevention of sexual assault by intervening when they witness its warning signs. In fact, in a contemporary “hookup culture” adolescents and young adults are more likely to meet and socialize in groups than they are to date in pairs and, therefore, warning signs of assault are frequently exhibited in communal spaces (Bogle, 2007; 2008; England & Ronen, 2015; Molidor & Tolman, 1998; Wade, 2017). Thus, the social nature of intimate relationships during these life stages can make peers pivotal actors in the prevention of sexual assault.

However, the potential for peer intervention can be undermined by a general “bystander effect” that diffuses responsibility for action in group settings (Darley & Latane, 1968). To intervene as a witness to sexual assault, individuals must notice the event (or its warning signs), define the event as warranting action/intervention, take responsibility for acting (i.e., feel a sense of personal duty), and demonstrate a sufficient level of self‐efficacy (i.e., perceived competence to successfully intervene; Latane & Darley, 1969). Studies have indicated that, as witnesses to sexual assault, young people often fail to meet these criteria (Banyard, 2008; Bennett, Banyard, & Garnhart, 2014; Burn, 2009; Casey & Ohler, 2012; Exner & Cummings, 2011; McCauley et al., 2013; McMahon, 2010; Noonan & Charles, 2009), with males being less likely than females to intervene (Banyard, 2008; Burn, 2009; Edwards, Rodenhizer‐Stampfli, & Eckstein, 2015; Exner & Cummings, 2011; McMahon, 2010).

Thus, bystander programs seek to sensitize young people to warning signs of sexual assault, create attitudinal change that fosters bystander responsibility for intervening (e.g., creating empathy for victims), and build requisite skills/tactics for taking action (Banyard et al., 2004; Banyard, 2011; Burn, 2009; McMahon & Banyard, 2012). Many of these programs are implemented with large groups of adolescents or college students in the format of a single training/education session (e.g., as part of college orientation). However, some programs use broader implementation strategies, such as advertising campaigns where signs are posted across college campuses to encourage students to act when witnessing signs of violence. The bystander model was developed and popularized in the US but has been adapted for use in global contexts.

### 6.3 How the intervention might work

By treating young people as potential allies in preventing sexual assault, bystander programs have the potential to be less threatening than traditional sexual assault prevention programs, which tend to approach young people as either potential perpetrators or victims of sexual violence (Burn, 2009; [Jackson] Katz, 1995; Messner, 2015). Instead of placing emphasis on how young people may modify their individual behavior to either respect the sexual boundaries of others or reduce their personal risk for being sexually assaulted, bystander programs aim to foster prerequisite knowledge and skills for intervening on behalf of victims. Thus, by treating young people as part of the solution to sexual assault, rather than part of the problem, bystander programs limit the risk of defensiveness or backlash among participants (e.g., decreased empathy for victims, increased rape myth acceptance; Banyard et al., 2004; Katz, 1995).

In addition to encouraging young people to prevent sexual violence through bystander intervention, these programs may reduce the likelihood of participants committing sexual assault themselves. To illustrate, Katz (1995) describes the rationale behind the mentors in violence prevention (MVP) bystander program as follows: “rather than focus on men as actual or potential *perpetrators* (emphasis in original), we focus on them in their role as potential *bystanders* (emphasis in original). This shift in emphasis greatly reduces the participants’ defensiveness” (p. 168). In other words, young men may be more receptive to prevention programming, and thus less likely to commit sexual assault, when they are approached as part of the solution rather than part of the problem to sexual assault. Consistent with this line of reasoning, studies on the effects of bystander programs often measure participants’ rates of sexual assault as a program outcome (Foubert, 2000; Foubert, Newberry, & Tatum, 2007; Gidycz, Orchowski, & Berkowitz, 2011; Miller et al., 2013; Miller et al., 2014; Salazar, Vivolo‐Kantor, Hardin, & Berkowitz, 2014).

As outlined by Burn (2009) bystander programs are designed to promote the following prerequisites for intervention: noticing an event, identifying a situation as warranting intervention, taking responsibility for acting, and deciding how to help. This often involves educating young people about what constitutes sexual assault, portraying victims as worthy of assistance, and building skills necessary to intervene (e.g., providing strategies for what to do and say). Although most bystander programs share the common goal of promoting such prerequisites for intervention, their specific program content and framing of sexual assault varies.

Research has indicated that, relative to males, females are overwhelmingly the victims of sexual assault (Foshee, 1996; Gressard, Swahn, & Tharp, 2015; Harned, 2001; Howard, Wang, & Yan, 2007). Thus, the earliest bystander programs tended to apply a gendered perspective to the prevention of sexual assault among adolescents and college students. For example, Katz (1995) developed MVP with the goal of inspiring male college athletes to challenge sociocultural definitions of masculinity that equate men's strength with dominance over women. At the time of its inception, MVP was unique in its explicit focus on masculinity as well as its nonthreatening bystander approach that encouraged young men to intervene when witnessing acts (or warning signs) of violence against women. As Katz explained, MVP reduces young men's defensiveness to violence prevention efforts by focusing on men as potential *bystanders to violence*, rather than potential *perpetrators of violence*. In addition to reducing men's defensiveness to intervention efforts, this bystander approach emphasizes the point that “when men don't speak up or take action in the face of other men's abusive behavior toward women, that constitutes implicit consent of such behavior” (Katz, 1995, p. 168).

Since the inception of MVP a number of programs have emerged to address barriers to bystander intervention among adolescents and college students. Although they all share the common goal of inspiring bystanders to act in ways that prevent sexual assault, these programs exhibit a great deal of variation in scope pertaining to their target bystander populations (i.e., males and/or females, secondary school or college students), sex of victims, and gendered versus gender‐neutral approach. For example, some programs use a gendered approach by (a) critiquing gender norms that can promote violence against women and (b) encouraging males to intervene on behalf of female victims (e.g., MVP, see Katz, 1995). Others use a gender‐neutral approach to build a sense of community responsibility to intervene on behalf of both male and female victims of sexual assault (e.g., Bringing in the Bystander, see Banyard, Moynihan, & Crossman, 2009; Banyard, Moynihan, & Plante, 2007). One of the major differences between gendered and gender‐neutral bystander programs is that the former places socio‐cultural forces, such as gender norms, at the center of discussions of violence whereas the latter places the bystander, and individual cognitive processes when encountering violence, at the center of discussions of violence ([Jackson] Katz, Heisterkamp, & Fleming, 2011; Messner, 2015).

Comparing the effects of gendered and gender‐neutral programs has the potential to identify important determinants of the success of bystander programs. Although there is no empirical examination of the different effects of these programs, there are theoretical reasons to believe that each has the potential to be successful under certain conditions. Namely, gendered approaches to bystander education programs may be better suited to target socio‐cultural facilitators of sexual assault against women and address different patterns of bystander behaviors exhibited by males and females (Banyard, 2008; Burn, 2009; Exner & Cummings, 2011; Katz, Heisterkamp, & Fleming, 2011; [Jennifer] Katz, 2015; [Jennifer] Katz, Colbert, & Colangelo, 2015; McCauley et al., 2013; McMahon, 2010; Messner, 2015). On the other hand, gender‐neutral programs may have the benefit of deflecting the criticism that prevention programs utilizing a gendered approach are inherently anti‐male (Katz et al., 2011; Messner, 2015). Proponents of such programs assert that avoidance of such criticism is paramount to the success of sexual assault prevention programs. In their view, adolescents and young adults who are coming of age in a “post‐feminist” era may be likely to reject gendered explanations of sexual assault and, instead, may respond more positively to gender‐neutral programs that use inclusive language that can be applied to a broad range of victims and perpetrators (Barreto & Ellemers, 2005; Kettrey, 2016; Swim, Aikin, Hall, & Hunter, 1995).

### 6.4 Why it is important to do the review

Policymakers in the United States perceive bystander programs to be beneficial, as evidenced by the 2013 Campus SaVE Act's requirement that postsecondary educational institutions participating in Title IX financial aid programs provide incoming college students with primary prevention and awareness programs addressing sexual violence. The Campus SaVE Act mandates that these programs include a component on bystander intervention. Currently, there is no comparable legislation regarding sexual assault among adolescents (e.g., mandating bystander programs in secondary schools). This is an unfortunate oversight, as adolescents who experience sexual assault are at an increased risk of repeated victimization in young adulthood (Cui et al., 2013). Thus, the implementation of bystander programs in secondary schools not only has the potential to reduce sexual assault among adolescents but may also have the long‐term potential to reduce sexual assault on college campuses.

Findings from this systematic review will provide valuable evidence of the extent to which bystander programs, as mandated by the Campus SaVE Act, are effective in preventing sexual assault among college students. Additionally, by examining effects of these programs among adolescents, this review will provide educators and policy makers with information for determining whether such programs should be widely implemented in secondary schools.

Currently, there are no Campbell or Cochrane Collaboration Reviews evaluating the effects of bystander programs on sexual assault among adolescents and/or college students. Of modest relevance to the proposed review, the Campbell and Cochrane Collaboration libraries include meta‐analyses of the effects of more general programs (not bystander programs) designed to prevent or reduce relationship/dating violence among adolescents and/or young adults (De La Rue, Polanin, Espelage, & Pigott, 2014; Fellmeth, Heffernan, Nurse, Habibula, & Sethi, 2013). Both of these reviews reported violence outcomes as aggregate measures that do not distinguish sexual violence from other forms of violence. Although they each found some evidence of significant effects on knowledge or attitudes pertinent to violence, neither found evidence of significant effects on young people's behavior (i.e., rates of perpetration or victimization).

Two reviews published outside of the Campbell and Cochrane Collaboration libraries are of closer relevance to this review. These include a meta‐analysis of the effects of bystander programs on sexual assault on college campuses (Katz & Moore, 2013) and a narrative review of studies examining the effects of bystander programs on dating and sexual violence among adolescents and young adults (Storer, Casey, & Herrenkohl, 2015).

In what they called an “initial” meta‐analysis of experimental and quasi‐experimental studies published through 2011, Katz & Moore (2013) found moderate beneficial effects of bystander programs on participants' self‐efficacy and intentions to intervene, and small (but significant) effects on bystander behavior, rape‐supportive attitudes, and rape proclivity (but not perpetration). Effects were generally stronger among younger samples and samples containing a higher percentage of males. The stronger effect for younger participants (i.e., younger college students) suggests such programs may be particularly effective with adolescents.

In a narrative review of studies examining the effects of bystander programs on dating violence and sexual assault among adolescents and young adults, Storer et al. (2015) highlighted beneficial effects on bystander self‐efficacy and intentions but noted less evidence of beneficial effects on actual bystander behavior or perpetration of violence. While informative, each of these reviews has limitations. Katz & Moore's (2013) meta‐analysis focused exclusively on sexual assault on college campuses and did not examine effects of such programs among adolescents. Although Storer et al. (2015) focused on studies examining violence among both adolescents and young adults, their sample was limited in that it was exclusively comprised of peer‐reviewed articles (i.e., the sample explicitly excluded theses, dissertations, and other gray literature). Additionally, the authors specified no research design criteria for inclusion (i.e., the sample included low‐quality studies such as those utilizing single group pre‐ and post‐test designs), limiting the strength of their conclusions. Importantly, Storer et al. reported no meta‐analytic findings. Thus, to our knowledge there are currently no existing meta‐analyses examining the effects of bystander programs on attitudes and behaviors regarding sexual assault among both college students and adolescents. Additionally, Katz & Moore's early meta‐analysis only included studies published/reported through 2011 (2 years prior to the 2013 Campus SaVE Act) and did not evaluate program content as a moderator.

Our review examined the effects of bystander programs on attitudes (i.e., perceptions of violence/victims, self‐efficacy to intervene, and intentions to intervene) and behaviors (i.e., actual intervention behavior, perpetration) regarding sexual assault among adolescents and college students. Importantly, we present meta‐analytic findings to quantitatively assess the influence of moderators (e.g., gender composition of sample, mean age of sample, education level of sample, single‐ or mixed‐sex implementation, gendered content of program, fraternity/sorority membership, and athletic team membership) on the effects of bystander programs.

## 7 OBJECTIVES

### 7.1 The problem, condition or issue

The overall objective of this systematic review and meta‐analysis was to examine the effects bystander programs have on preventing sexual assault among adolescents and college students. More specifically, and given the study designs that were included, this review addressed three objectives.

1. The first objective was to assess the overall effects (including adverse effects), and the variability of the effects, of bystander programs on adolescents' and college students' attitudes and behaviors regarding sexual assault. This included general attitudes toward violence and victims, prerequisite skills and knowledge to intervene, self‐efficacy to intervene, intentions/willingness to intervene when witnessing signs of sexual assault, actual intervention behavior, and perpetration of sexual assault.
2. The second objective was to explore the comparative effectiveness of bystander programs for different profiles of participants (e.g., mean age of the sample, education level of the sample, proportion of males/females in the sample, proportion of fraternity/sorority members in the sample, and proportion of athletic team members in the sample).
3. The third objective was to explore the comparative effectiveness of different bystander programs in terms of gendered content and approach (e.g., conceptualizing sexual assault as a gendered or gender‐neutral problem, mixed‐ or single‐sex group implementation).

## 8 METHODS

### 8.1 Criteria for considering studies for this review

#### 8.1.1 Types of studies

To be eligible for inclusion in the review, studies must have used an experimental or controlled quasi‐experimental research design to compare an intervention group (i.e., students assigned to a bystander program) with a comparison group (e.g., students not assigned to a bystander program). We limited our review to such study designs because these typically have lower risk of bias relative to other study designs (e.g., single group designs). More specifically, we included the following designs:

1. Randomized controlled trials: Studies in which individuals, classrooms, schools, or other groups were randomly assigned to intervention and comparison conditions.
2. Quasi‐randomized controlled trials: Studies where assignment to conditions was quasi‐random, for example, by birth date, date of week, student identification number, month, or some other alternation method.
3. Controlled quasi‐experimental designs: Studies where participants were not assigned to conditions randomly or quasi‐randomly (e.g., participants self‐selected into groups). Given the potential selection biases inherent in these controlled quasi‐experimental design, we only included those that also met one of the following criteria:
  a. Regression discontinuity designs: Studies that used a cutoff on a forcing variable to assign participants to intervention and comparison groups, and assessed program impacts around the cutoff of the forcing variable.
  b. Studies that used propensity score or other matching procedures to create a matched sample of participants in the intervention and comparison groups. To be eligible for inclusion, these studies must have also provided enough statistical information to permit estimation of pretest effect sizes for the matched groups.
  c. For studies where participants in the intervention and comparison groups were not matched, enough statistical information must have been reported to permit estimation of pretest effect sizes for at least one outcome measure.

Consistent with Campbell Collaboration policies and procedures, studies using experimental and quasi‐experimental research designs were synthesized separately in the meta‐analyses, given that experimental study designs have the highest level of internal validity. Furthermore, we collected extensive data on the risk of bias and study quality of all eligible studies, which we attended to when interpreting the findings from the systematic review and meta‐analyses (described in greater detail below).

#### 8.1.2 Types of participants

The review focused on studies that examined outcomes of bystander programs and target sexual assault and are implemented with adolescents and/or college students in educational settings. Eligible participants included adolescents enrolled in grades 7 through 12 and college students enrolled in any type of undergraduate postsecondary educational institution.

Eligible participant populations included studies that reported on general samples of adolescents and/or college students as well as studies using specialized samples such as those primarily consisting of college athletes, fraternity/sorority members, and single‐sex samples. Study samples primarily consisting of postgraduate students were ineligible for inclusion; the mean age of samples could be no less than 12 and no greater than 25 to be included in the review.

#### 8.1.3 Types of interventions

Eligible intervention programs were those that approached participants as allies in preventing and/or alleviating sexual assault among adolescents and/or college students. Some part of the program had to focus on ways that cultivate willingness for a person to respond to others who are at risk for sexual assault. All delivery formats were eligible for inclusion (e.g., in‐person training sessions, video programs, web‐based training, and advertising/poster campaigns). There were no interventions duration criteria for inclusion.

Studies that reported bystander outcomes but did not meet the aforementioned intervention inclusion criterion were not eligible for inclusion. Additionally, studies that assessed outcomes of programs that aimed to facilitate pro‐social bystander behavior, but that did not explicitly include a component addressing sexual assault (e.g., programs to prevent bullying) were not eligible for inclusion.

Eligible comparison groups must have received no intervention services targeting bystander attitudes/behavior or sexual assault. Thus, treatment–treatment studies that compared outcomes of individuals assigned to a bystander program versus those assigned to a general sexual assault prevention program were not eligible for inclusion. Eligible comparison groups may have received a sham or attention treatment expected to have no effect on bystander outcomes or attitudes/behaviors regarding sexual assault.

#### 8.1.4 Types of outcome measures

We included studies that measured the effects of bystander programs on at least one of the following primary outcome domains:

1. General attitudes toward sexual assault and victims (e.g., victim empathy, rape myth acceptance).
2. Prerequisite skills and knowledge for bystander intervention as defined by Burn (2009) (e.g., noticing sexual assault or its warning signs, identifying a situation as appropriate for intervention, taking responsibility for acting/intervening, knowing strategies for helping/intervening).
3. Self‐efficacy with regard to bystander intervention (e.g., respondents' confidence in their ability to intervene).
4. Intentions to intervene when witnessing instances or warning signs of sexual assault.
5. Actual intervention behavior when witnessing instances or warning signs of sexual assault.
6. Perpetration of sexual assault (i.e., rates of perpetration among individuals assigned to the treatment or comparison group of a study).

Depending on directionality, these outcomes capture both beneficial and adverse effects (e.g., increases or decreases in victim empathy, pro‐social bystander behavior, etc.) that are important to adolescents, college students, and decision‐makers alike.

Any outcome falling in these domains was eligible for inclusion. This includes outcomes measured with any form of assessment (e.g., self‐report, official/administrative report, observation, etc.) that could be summarized by any type of quantitative score (e.g., percentage, continuous variable, count variable, categorical variable, etc.). In the event that a particular study included multiple measures of a single construct category (e.g., two measures of rape myth acceptance or bystander intervention within a given study), we only included one outcome per study for that construct. We selected the most similar outcomes for synthesis within a construct category (described in detail below).

#### 8.1.5 Duration of follow‐up

Studies reporting follow‐ups of any duration were eligible for inclusion. When studies reported more than one follow‐up wave, each wave was coded and identified by its reported duration. As described in more detail below, follow‐ups of similar durations were analyzed together.

#### 8.1.6 Types of settings

The review focused on studies that examine outcomes of bystander programs and target sexual assault and are implemented with adolescents and/or college students in educational settings. Eligible educational settings included secondary schools (i.e., grades 7–12) and colleges or universities. Studies that assessed bystander programs implemented with adolescents and young adults outside of educational institutions (e.g., in community settings, military settings) were ineligible for inclusion in the review. There were no geographic limitations on inclusion criteria. Research conducted in any country was eligible.

### 8.2 Search methods for identification of studies

#### 8.2.1 Search strategy

We identified candidate studies through searches of electronic databases, relevant academic journals, and gray literature sources. We also contacted leading authors and experts on bystander programs to identify any current/ongoing research that might be eligible for the review. Additionally, we screened the bibliographies of eligible studies and relevant reviews to identify additional candidate studies. We conducted forward citation searches (searches for reports citing eligible studies) using the website Google Scholar, as this database produces similar results to other search engines (e.g., Web of Science; Tanner‐Smith & Polanin, 2015) and is also more likely to locate existing gray literature. Our search was global in scope and attempted to identify studies of bystander programs implemented in any country.

#### 8.2.2 Electronic searches

The prevention of sexual assault among college students and adolescents is a topic that spans multiple disciplines (e.g., sociology, psychology, education, and public health). Thus, we searched a variety of databases that are relevant to these fields. Search terms varied by database, but generally included two blocks of terms and appropriate Boolean or proximity operators, when allowed: blocks included terms that address the intervention and outcomes. We specifically searched the following electronic databases (hosts) in October 2016 and June 2017:

- Cochrane Central Register of Controlled Trials (CENTRAL).
- Cochrane Database of Abstracts of Reviews of Effects (DARE).
- Education Resources Information Center (ERIC, via ProQuest).
- Education Database (via ProQuest).
- International Bibliography of the Social Sciences (IBSS, via ProQuest).
- PsycINFO (via ProQuest).
- PsycARTICLES (via ProQuest).
- PubMed.
- Social Services Abstracts (via ProQuest).
- Sociological Abstracts (via ProQuest).

The strategy for searching electronic databases involved the use of search terms specific to the types of interventions and outcomes eligible for inclusion. Search terms for types of interventions included general terms for bystander programs as well as names of specific bystander programs (e.g., MVP). Search terms for types of outcomes included terms that are specific to measures of sexual violence (e.g., sexual assault) as well as more general terms that have the potential to identify studies that measure physical and/or sexual violence. Due to the overwhelming focus of bystander programs on adolescents and college students (aside from a few implementations with military samples) search terms did not limit initial results by the age or general characteristics of the target population.

The search terms and strategy for PsycINFO via ProQuest were as follows (terms were modified for other databases):

(AB,TI(“bystander”)) AND (AB,TI(“education” OR “program” OR “training” OR “intervention” OR “behavior” OR “attitude” OR “intention” OR “efficacy” OR “prosocial” OR “pro‐social” OR “empowered” OR “Bringing in the Bystander” OR “Green Dot” OR “Step Up” OR “Mentors in Violence Prevention” OR “MVP” OR “Know Your Power” OR “Hollaback” OR “Circle of 6” OR “That's Not Cool” OR “Red Flag Campaign” OR “Where Do You Stand” OR “White Ribbon Campaign” OR “Men Can Stop Rape” OR “The Men's Program” OR “The Women's Program” OR “The Men's Project” OR “Coaching Boys into Men” OR “Campus Violence Prevention Program” OR “Real Men Respect” OR “Speak Up Speak Out” OR “How to Help a Sexual Assault Survivor” OR “InterACT”)) AND (AB,TI(“sexual assault” OR “rape” OR “violence” OR “victimization”))

#### 8.2.3 Searching other resources

We searched the tables of contents of current issues of journals that publish research on sexual violence. This included the following journals: *Journal of Adolescent Health, Journal of Community Psychology, Journal of Family Violence, Journal of Interpersonal Violence, Psychology of Violence, Violence Against Women,* and *Violence and Victims.* We searched these sources in October 2016 and June 2017.

We also conducted gray literature searches to identify unpublished studies that met inclusion criteria. This included searching electronic databases that catalog dissertations and theses, searching conference proceedings, and searching websites with content relevant to sexual assault and/or violence against women. We specifically searched the following gray literature sources in October 2016 and June 2017:

- ProQuest Dissertations and Theses Global.
- Clinical Trials Register (https://clinicaltrials.gov).
- End Violence Against Women International ‐ conference proceedings (http://www.evawintl.org/conferences.aspx).
- National Sexual Violence Resource Center website (nsvrc.org).
- National Online Resource Center on Violence Against Women website (VAWnet.org).
- Us Department of Justice Office on Violence Against Women website (www.justice.gov/ovw).
- Center for Changing our Campus Culture website (www.changingourcampusculture.org).

Additionally, we searched reference lists of previous systematic reviews and meta‐analyses, CVs and websites of primary authors of eligible studies, and reference lists of eligible studies. We also conducted forward citation searches of all eligible studies.

### 8.3 Data collection and analysis

#### 8.3.1 Selection of studies

Once candidate studies were identified in the literature search, each reference was entered into the project database as a separate record. Two reviewers then independently screened each study title and abstract and recorded their eligibility recommendation (i.e., ineligible or eligible for full‐text screening) into the pertinent database record. Disagreements between reviewers were resolved by discussion and consensus, and the final abstract screening decision was recorded in the database. Potentially eligible studies were then retrieved in full text and these full texts were reviewed for eligibility, again using two independent reviewers who recorded their eligibility recommendation (and, when applicable, rationale for an ineligibility recommendation). Disagreements between reviewers were again resolved via discussion and consensus and the final eligibility decision was recorded in the database. In cases where we could not determine eligibility due to missing information in a report, we contacted study authors for this information.

Throughout the search and screening process we maintained a document that included the number of unique candidate studies identified through various sources (e.g., electronic database searches, academic journal searches, and gray literature searches). We used the information in this record to create a PRISMA flow chart that reports the screening process (Moher et al., 2015). Additionally, we used the final screening decisions recorded in the meta‐analysis database to create a table that lists studies excluded during the full‐text screening phase along with the rationale for each exclusion decision.

#### 8.3.2 Data extraction and management

Two reviewers independently double‐coded all included studies, using a piloted codebook (see Appendix). All coding was entered into an electronic database, with a separate record maintained for each independent coding of each study. Coding disagreements were resolved via discussion and consensus with final coding decisions maintained in a separate record. If data needed to calculate an effect size were missing from a report, we contacted the primary study authors for this information.

The primary categories for coding are as follows: participant demographics and characteristics (e.g., age, gender, education level, race/ethnicity, athletic team membership, fraternity/sorority membership); intervention setting (e.g., state, country, secondary or postsecondary institution, mixed‐ or single‐sex group); study characteristics (e.g., attrition, duration of follow‐up, study design, participant dose, sample *N*); outcome construct (e.g., type, description of measure); and outcome results (e.g., timing at measurement, baseline and follow‐up means and standard deviations or proportions).

#### 8.3.3 Assessment of risk of bias in included studies

We assessed risk of bias in included studies using the Cochrane risk of bias tools for randomized studies (Higgins & Green, 2011) and nonrandomized studies (Sterne, Higgins, & Reeves, 2016). The Cochrane risk of bias tool for randomized studies assesses risk of bias in the following domains: random sequence generation, allocation concealment, blinding of participants and personnel, blinding of outcome assessment, incomplete outcome data, selective reporting, and other bias. These domains are each rated as low, unclear, or high risk of bias.

The Cochrane risk of bias tool for nonrandomized studies, ROBINS‐I, requires that at the protocol stage of the review, two sets of items are determined: (a) confounding areas that are expected to be relevant to all or most studies in the review and (b) cointerventions that could be different between intervention groups with the potential to differentially impact outcomes. In our protocol, we anticipated the following confounding factors, which were coded in the risk of bias assessments for nonrandomized studies: gender, fraternity/sorority membership, athletic team membership, preintervention attitudes (e.g., victim empathy, rape myth acceptance), preintervention bystander measures (e.g., efficacy, intentions, behavior), and prior sexual assault victimization. Additionally, we anticipated the following cointerventions to have a potential impact on outcomes, and they were coded in the risk of bias assessments: general sexual assault prevention programs, dating violence prevention programs, and general bystander programs (not explicitly targeting sexual violence).

#### 8.3.4 Measures of treatment effect

We extracted relevant summary statistics (e.g., means and standard deviations, proportions, observed sample sizes) to calculate effect sizes. We then used Wilson's (2013) online effect size calculator to calculate effect sizes. The overwhelming majority of studies reported continuous measures of treatment effects, so we used a SMD effect size metric with a small sample correction (i.e., Hedges' *g*). In the rare cases in which binary outcome measures were reported in included studies, we deemed these measures to represent the same underlying construct as continuous measures, as they typically relied on the same measurement tools as relevant continuous measures. Thus, we transformed any log odd ratio effect sizes available from binary measures into SMD effect sizes by entering the observed proportions and sample sizes into Wilson's (2013) online effect size calculator. All SMD effect sizes were coded such that positive values (i.e., greater than 0) indicate a beneficial outcome for the intervention group.

#### 8.3.5 Unit of analysis issues

The unit of analysis of interest for this review was the individual (i.e., individual‐level attitudes and behaviors). Nine of the included studies used cluster randomized trial designs where participants were randomized into the intervention or comparison conditions at the group level (e.g., entire schools were assigned to a single condition), and inferences were made at the individual level. To correct for these unit of analysis errors, we followed the procedures outlined in the Cochrane Handbook (Higgins & Green, 2011) to inflate the standard errors of the effect sizes from these nine studies by multiplying them by the design effect:

1+(M−1)ICC,

where *M* is the average cluster size for a given study and ICC is the intracluster correlation coefficient for a given outcome. In cases where study authors did not report ICCs we used a liberal assumed value of 0.10. This value has been used in a past Campbell review that synthesizes attitude and behavior outcomes of dating/sexual violence prevention programs (De La Rue et al., 2014) and is supported by Hedges and Hedberg's (2007) research on ICCs in cluster randomized trials conducted in educational settings.

#### 8.3.6 Dealing with missing data

When studies reported insufficient data to calculate effect sizes we contacted the primary authors to request the necessary information. This author query method was highly successful; there were only two cases in which we were unable to obtain sufficient data from study authors (see details below with search results).

#### 8.3.7 Assessment of heterogeneity

We assessed and reported heterogeneity using the *χ* ^2^ statistic and its corresponding *p* value, as well as the *I* ^2^ and *τ* ^2^ statistics. We used the restricted maximum likelihood estimator for *τ* ^2^. When at least 10 studies were included in any given meta‐analysis, we also used mixed‐effect meta‐regression models to conduct moderator analyses. Moderators specified in the protocol included: (a) gendered content of the program, (b) mixed‐ or single‐sex group implementation, (c) gender composition of the sample, (d) education level of the sample (i.e., secondary school or college students), (e) mean age of the sample, (f) proportion of fraternity/sorority members in the sample, and (g) and proportion of athletic team members in the sample.

#### 8.3.8 Assessment of reporting biases

To assess the potential of small study/publication bias, we used the contour enhanced funnel plot (Palmer, Peters, Sutton, & Moreno, 2008), Egger's regression test (Egger, Smith, Schneider, & Minder, 1997), and trim and fill analysis (Duval & Tweedie, 2000).

#### 8.3.9 Data synthesis

We conducted all statistical analyses with the metafor package in *R*. We conducted meta‐analyses using random‐effects inverse variance weights and reported 95% confidence intervals along with all mean effect size estimates. We conducted and reported all meta‐analyses separately for RCTs and non‐RCTs. Additionally, we conducted all syntheses separately by outcome domain (i.e., attitudes toward sexual assault and victims, prerequisite skills and knowledge to intervene, bystander self‐efficacy, bystander intentions, bystander behavior, perpetration) and follow‐up timing. For most outcomes, follow‐up timing fell into the following categories: immediate post‐intervention, 1‐ to 4‐month post‐intervention, and 6 months to 1‐year post‐intervention. We displayed each synthesis using forest plots.

To minimize any potential bias in the meta‐analysis results due to effect size outliers, prior to conducting the meta‐analysis, we Winsorized any outlier effect sizes that fell more than two standard deviations away from the mean of the effect size distribution (Lipsey & Wilson, 2001). In such cases, we replaced the outlier effect size with the value that fell exactly two standard deviations from the mean of the distribution of effect sizes.

As noted previously, each meta‐analysis synthesized a set of statistically independent effect sizes. To ensure the statistical independence of effect sizes synthesized within any given meta‐analysis, we split all analyses by follow‐up timing and outcome domain. In the event that a particular study included multiple measures within a single outcome domain (e.g., two measures of rape myth acceptance within a given study), we only included one outcome per study for that construct. We selected the most similar outcomes for synthesis within a construct category. We adopted this approach for synthesizing statistically independent effect sizes given the small number of included studies and effect sizes available for synthesis. Other approaches that can be used to synthesize dependent effect sizes, such as robust variance estimation, require larger sample sizes for efficient parameter estimation than the sample sizes we had available for analysis (Hedges, Tipton, & Johnson, 2010; Tanner‐Smith & Tipton, 2014; Tipton, 2013).

#### 8.3.10 Subgroup analysis and investigation of heterogeneity

We performed sub‐group analyses based on study design, synthesizing effect sizes (a) for RCTs alone, (b) for non‐RCTs alone, and (c) for RCTs and non‐RCTS combined. For each of these three subgroup analyses we assessed and reported heterogeneity using the *χ* ^2^ statistic and its corresponding *p* value, *I* ^2^, and *τ* ^2^. When at least ten studies were included in any given meta‐analysis, we used mixed‐effect meta‐regression models to conduct moderator analyses.

#### 8.3.11 Sensitivity analysis

When at least 10 studies were included in a given meta‐analysis we conducted sensitivity analyses to examine whether study‐level attrition and high risk of bias (for each domain assessed with the risk of bias tools) were associated with effect size magnitude, using mixed‐effects meta‐regression models. Additionally, we conducted sensitivity analyses that removed any Winsorized effect sizes from the meta‐analysis, to assess whether this method for handling outliers may have substantively altered the review findings.

#### 8.3.12 Interpretation of findings

To provide substantive interpretations of statistically significant mean effect sizes, we transformed the average SMD effect size back into an unstandardized metric using commonly reported scales/measures in the respective outcome domains. Namely, we multiplied the average SMD effect size by the standard deviation of a scale/measure that was frequently used in our meta‐analytic sample to yield an unstandardized difference in means. We aimed to select means and standard deviations from the most representative studies in our sample (e.g., RCTs and/or studies with larger sample sizes), but we recognize this is an imperfect process that requires generalization from a single study (for each significant outcome). We present these transformations in our Discussion section, at the conclusion of this report. Readers should be mindful of the fact that these are extrapolations intended to provide meaningful context to our findings, and that results are most accurately represented by the Hedges' *g* effect sizes.

## 9 RESULTS

### 9.1 Description of studies

#### 9.1.1 Results of the search

**Figure 1:**
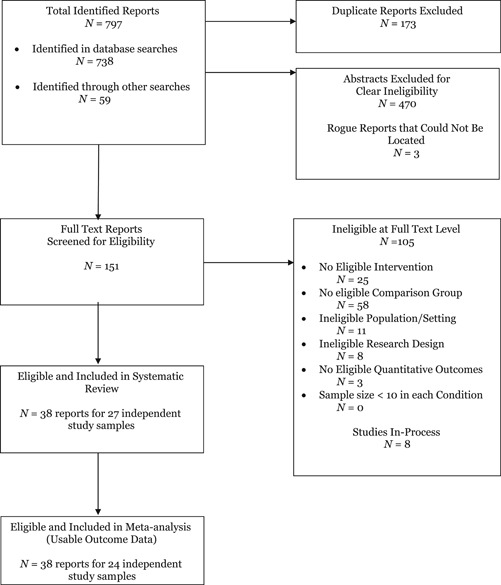
PRISMA study flow diagram

We conducted an initial literature search in October 2016 and an updated search in June 2017. Figure 1 outlines the flow of studies through the search and screening process. Through our initial and updated search we identified 797 reports. Of these reports, 738 were identified from searches of electronic databases, 19 from ClinicalTrials.gov, 1 from conference proceedings, 5 from website searches, 20 from reference lists of review articles, 1 from tables of contents searches, 3 from reference lists of eligible reports, 9 from CVs and websites of primary authors of eligible studies, and 1 from forward citation searching of eligible studies. After deleting duplicate reports and reports that were deemed ineligible through the abstract screening process 154 reports were deemed eligible for full‐text screening. Three of these reports could not be located; thus, we screened a total of 151 full‐text reports for eligibility.

#### 9.1.2 Included studies

Twenty‐seven independent studies summarized in 38 reports met inclusion criteria. One report (Jouriles, Rosenfield, Yule, Sargent, & McDonald, 2016b) presented findings from two independent studies. We coded these two studies separately and identified them as Jouriles et al. (2016a) and Jouriles et al. (2016b). Table 1 summarizes aggregate characteristics of all eligible studies. Twelve studies utilized random assignment at the individual level, nine studies utilized random assignment at the group level, and six studies utilized nonrandom assignment but reported pretest equivalence measures on at least one eligible outcome. More specific details of each individual study are summarized in Table A1 (see Figures and Tables Appendix). The majority of studies were conducted in the United States, with one study conducted in Canada and one conducted in India. This geographic pattern was primarily due to a paucity of research on bystander programs conducted outside of the United States and, to a lesser extent, to the failure of studies conducted outside the United States to meet the scope and methodological criteria for this review.

**Table 1:** Characteristics of included studies (N = 27)

	*n*	%
**Study design**		
Randomized – Individual	12	44.44
Randomized – Group	9	33.33
Nonrandomized	6	22.22
**Comparison treatment**		
Active/Sham treatment	9	33.33
Inactive control	18	66.67
**Peer reviewed**	22	81.48
**Funded**	18	66.67
**Study report year**		
2017	2	7.41
2016	6	22.22
2015	4	14.81
2014	4	14.81
2012	1	3.70
2011	2	7.41
2010	1	3.70
2008	1	3.70
2007	2	7.41
2000	1	3.70
1998	1	3.70
1997	1	3.70
Not reported	1	3.70
**Educational setting**		
College/University	22	81.48
Secondary school	5	18.52
**Country**		
United States	25	92.59
Canada	1	3.70
India	1	3.70

Three studies were eligible for the review by methodological standards but did not report codable outcomes (Cook‐Craig et al. 2014; Darlington, 2014; Mabry & Turner, 2016). For example, in Cook‐Craig et al.'s (2014) study the unit of analysis was the school, not the individual. The study authors surveyed entire intervention and comparison schools across several years. As a result, the individual students composing the sample changed from wave to wave—and many students entering the school in later waves did not receive direct intervention. Darlington (2014) only reported within‐group pre‐post effect sizes. No between‐group data were reported and our attempt to obtain these data from the study author was unsuccessful. Mabry and Turner (2016) reported three‐way ANOVA findings from a three‐armed study (i.e., one eligible intervention group, one ineligible intervention group, and a comparison group). Our attempts to obtain data for the eligible intervention and comparison groups from the study author were unsuccessful. We therefore summarized each of these three studies in the systematic review but were unable to include results from those studies in any of the meta‐analyses.

Two studies (Banyard et al., 2007; Jouriles et al., n.d.) included two eligible intervention arms. Banyard et al. (2007) randomly assigned participants to one of three groups: (a) a no‐treatment control that only completed pre‐ and post‐intervention surveys, (b) an intervention group that was assigned to complete one 90‐min bystander program session, or (c) an intervention group that was assigned to complete three 90‐min bystander program sessions. Jouriles et al. (n.d.) randomly assigned participants to one of three groups: (a) a comparison “sham treatment” condition that presented material on study skills, (b) a computer‐delivered bystander program that participants completed independently, or (c) a computer‐delivered bystander program that participants completed in a lab under supervision. We handled these multiple‐arm studies by selecting the intervention groups that were most consistent with the intervention groups from the other studies in the sample (i.e., the single‐session treatment for Banyard et al. and the unmonitored treatment for Jouriles et al.). We contrasted these intervention groups with their comparison groups and included the calculated effect sizes in our main meta‐analyses. We then conducted sensitivity analyses in which we ran additional meta‐analyses that included effect sizes that compared the alternative intervention arms with the comparison arm.

#### 9.1.3 Excluded studies

As shown in Figure 1, 105 reports were deemed ineligible after full‐text screening. These reports were ineligible because they either did not present an evaluation of an eligible intervention (*n* = 25), did not include an eligible comparison group (*n* = 58), did not involve an eligible population or setting (*n* = 11), did not involve an eligible research design (*n* =8), or did not measure any eligible outcomes (*n* = 3). Table A2 provides a brief description of each of these studies as well as the specific reasons for exclusion (see Figures and Tables Appendix).

### 9.2 Risk of bias in included studies

#### 9.2.1 Randomized studies

Table 2 summarizes the risk of bias for the 21 randomized studies included in the systematic review. A large percentage of studies failed to report information that would permit assessment of risk of bias; these were coded as unclear risk. Among those studies reporting sufficient information, most exhibited low risk of bias in the domains of random sequence generation, allocation concealment, blinding of participants, and selective reporting. The majority of randomized studies (95.2%) exhibited high risk of bias in blinding of outcome assessment; these studies relied on self‐report outcome data. Although a slight majority of randomized studies (52.4%) exhibited low risk of bias in handling incomplete outcome data, one‐third exhibited high risk of bias. These studies with high risk of bias tended to exhibit uneven attrition between the intervention and comparisons groups or handled missing data inappropriately (e.g., mean imputation). The majority of randomized studies (81.0%) exhibited high risk of bias in the “other” domain; these studies tended to be conducted by researchers who either developed the intervention under evaluation or were closely affiliated with the developer of the intervention under evaluation.

**Table 2:** Risk of bias of randomized studies

	Low risk		High risk		Unclear risk	
	*n*	%	*n*	%	*n*	%
Random sequence generation	8	38.1	0	0	13	61.9
Allocation concealment	3	14.3	0	0	18	85.7
Blinding of participants	1	4.8	0	0	20	95.2
Blinding of outcome assessment	1	4.8	20	95.2	0	0
Incomplete outcome data	11	52.4	7	33.3	3	14.3
Selective reporting	19	90.5	2	9.5	0	0
Other bias	3	14.3	17	81.0	1	4.8

*Note: N* = 21 studies.

#### 9.2.2 Nonrandomized studies

Table 3 summarizes the risk of bias for the six nonrandomized studies included in the systematic review. Among the studies that reported group differences in confounding variables, most reported congruence between groups. This was often a product of the targeted population. For example, three studies targeted a specific gender (i.e., young men or young women) and two specifically targeted fraternity or sorority members. Only one study reported group differences between participants' previous experiences with co‐interventions. The remaining studies either failed to report this information (*n* = 3) or reported it at the aggregate level (*n* = 2).

**Table 3:** Risk of bias of nonrandomized studies

	Even between groups		Uneven between groups		Not reported	
	*n*	%	*n*	%	*n*	%
Confounding variables						
Gender	4	66.7	1	16.7	1	16.7
Fraternity/sorority	2	33.3	0	0	4	66.7
Athlete	1	16.7	0	0	5	83.3
Preintervention attitudes	3	50.0	0	0	3	50.0
Prior victimization	0	0	1	16.7	5	83.3
Confounding interventions	0	0	1	16.7	5	83.3

*Note: N* = 6 studies.

### 9.3 Synthesis of results

#### 9.3.1 Victim empathy

Two studies measured victim empathy as an intervention outcome (Brokenshire, 2015; Salazar et al., 2014). Both studies operationalized victim empathy using the Rape Empathy Scale (Deitz, Blackwell, Daley, & Bentley, 1982), which asks participants to rate their agreement with statements such as “In general, I feel that rape is an act that is not provoked by the rape victim.” Both of these studies involved random assignment of participants to conditions at the individual level. Salazar et al. was published in a peer‐reviewed journal and Brokenshire was an unpublished master's thesis. Because the two studies measured the outcome at very different time points, we could not synthesize effect sizes. Brokenshire measured victim empathy as an immediate posttest outcome (*g* = −0.44, 95% CI [−0.79, −0.09]) and Salazar et al. reported victim empathy 6 months post‐intervention (*g* = 0.68, 95% CI [0.41, 0.95]). Notably, effect sizes from these two studies are divergent, with Brokenshire indicating a significant negative intervention effect at immediate posttest and Salazar et al. indicating a significant positive effect 6 months post‐intervention.

#### 9.3.2 Rape myth acceptance

Twelve studies measured rape myth acceptance as an intervention outcome. These studies most often operationalized rape myth acceptance using the Illinois Rape Myth Acceptance Scale (Payne, Lonsway, & Fitzgerald, 1999), which asks respondents to rate their agreement with statements such as, “rape happens when a man's sex drive gets out of control.” All but four of these studies (Amar, Tuccinardi, Heislein, & Simpson, 2015; Baker, Naai, Mitchell, & Trecker, 2014; Foubert & Marriott, 1997; Peterson et al., 2016) involved random assignment of participants to conditions. All but one study (Brokenshire, 2015) was published in a peer‐reviewed outlet; this study was an unpublished master's thesis. We collapsed bystander efficacy effect sizes into three follow‐up intervals: (a) immediate posttest (i.e., 0 week to 1 week), (b) 1 month to 4 months post‐intervention, and (c) 6 to 7 months post‐intervention.

##### 9.3.2.1 Immediate post‐test effects

**Figure 2:**
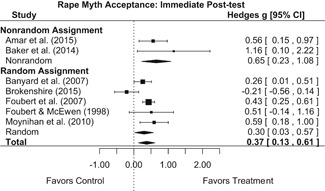
Forest plot of bystander intervention effects on rape myth acceptance – immediate posttest

Seven studies reported rape myth acceptance as an immediate posttest outcome. As shown in the forest plot in Figure 2, the intervention effect for the two nonrandomized studies was significant and positive (*g* = 0.65, 95% CI [0.23, 1.08]) with minimal between‐study heterogeneity (*χ* ^2^ = 1.07 [*p* = .30], *I* ^2^ = 6.75%, *τ* ^2^ = 0.01). The intervention effect for the five randomized studies was also significant and positive (*g* = 0.30, 95% CI [0.03, 0.57]), with significant between‐study heterogeneity (*χ* ^2^ = 12.35 [*p* = .01], *I* ^2^ = 71.90%, *τ* ^2^ = 0.06). When we synthesized the findings from the randomized and nonrandomized studies together, the average intervention effect was significant and positive (*g* = 0.37, 95% CI [0.13, 0.61]) with significant between‐study heterogeneity (*χ* ^2^ = 15.73 [*p* = .02], *I* ^2^ = 66.36%, *τ* ^2^ = 0.06). Thus, at immediate post‐intervention, the results indicate that bystander programs have a positive (beneficial) effect on rape myth acceptance. However, the small sample size in this meta‐analysis (*n* < 10) precluded ad hoc analysis of moderators or small study/publication bias.

**Figure 3:**
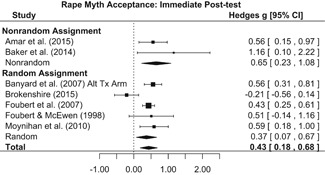
Forest plot of bystander intervention effects on rape myth acceptance—immediate posttest with alternative Tx arm for Banyard et al. (2007)

Sensitivity analysis in which the Banyard et al. (2007) one‐session intervention was replaced by the three‐session intervention did not substantively change these findings. As illustrated in Figure 3, the treatment effect for the five randomized studies (which included Banyard et al., 2007) was significant and positive (*g* = 0.37, 95% CI [0.07, 0.67]) with significant between‐study heterogeneity (*χ* ^2^ = 14.04 [*p* = .01], *I* ^2^ = 76.48%, *τ* ^2^ = 0.08). When synthesizing all studies together, the average intervention was significant and positive (*g* = 0.43, 95% CI [0.18, 0.68]) with significant between‐study heterogeneity (*χ* ^2^ = 16.47 [*p* = .01], *I* ^2^ = 68.55%, *τ* ^2^ = 0.07).

##### 9.3.2.2 One to four month post‐intervention effects

**Figure 4:**
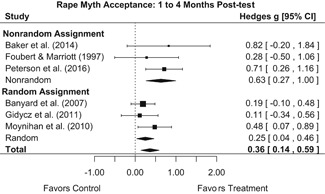
Forest plot of bystander intervention effects on rape myth acceptance—1 to 4 months post‐intervention

Six studies reported rape myth acceptance 1 to 4 months post‐intervention. As depicted in the forest plot in Figure 4 the intervention effect for the three nonrandomized studies was significant and positive (*g* = 0.63, 95% CI [0.27, 1.00]) with nonsignificant heterogeneity (*χ* ^2^ = 1.02 [*p* = .60], *I* ^2^ = 0.00%, *τ* ^2^ = 0). The intervention effect for the three randomized studies was significant and positive (*g* = 0.25, 95% CI [0.04, 0.46]) with nonsignificant heterogeneity (*χ* ^2^ = 1.73 [*p* = .42], *I* ^2^ = 0.00%, *τ* ^2^ = 0). Across all studies, the average intervention effect was significant and positive (*g* = 0.36, 95% CI [0.14, 0.59]) with nonsignificant heterogeneity (*χ* ^2^ = 5.90 [*p* = .32], *I* ^2^ = 23.73%, *τ* ^2^ = 0.02). This average intervention effect indicates that, at 1‐ to 4‐month post‐intervention, bystander programs have a positive (beneficial) effect on rape myth acceptance. However, the small sample size in this meta‐analysis (*n* < 10) precluded ad hoc analysis of moderators or small study/publication bias.

**Figure 5:**
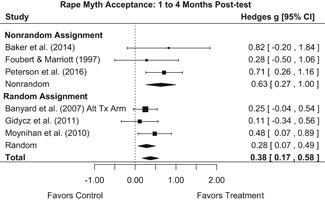
Forest plot of bystander intervention effects on rape myth acceptance—1 to 4 months post‐intervention—with alternative Tx arm for Banyard et al. (2007)

Sensitivity analysis in which the Banyard et al. (2007) one‐session intervention was replaced by the three‐session intervention did not substantively change these findings. As illustrated in Figure 5, the treatment effect for the five randomized studies (which included Banyard et al., 2007) was significant and positive (*g* = 0.28, 95% CI [0.07, 0.49]) with nonsignificant heterogeneity (*χ* ^2^ = 1.49 [*p* = .47], *I* ^2^ = 0.00%, *τ* ^2^ = 0). Across all studies, the average intervention effect (for randomized and nonrandomized studies) was significant and positive (*g* = 0.38, 95% CI [0.17, 0.58]) with nonsignificant heterogeneity (*χ* ^2^ = 5.18 [*p* = .39], *I* ^2^ = 12.40%, *τ* ^2^ = 0.01).

##### 9.3.2.3 Six to seven month post‐intervention effects

**Figure 6:**
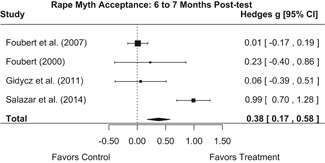
Forest plot of bystander intervention effects on rape myth acceptance—6 to 7 months post‐intervention

Four studies reported rape myth acceptance outcomes at 6 to 7 months post‐intervention. Each of these studies involved random assignment of participants to conditions. As shown in the forest plot in Figure 6, the average intervention effect across these four studies was significant and positive (*g* = 0.38, 95% CI [0.17, 0.58]) with nonsignificant between‐study heterogeneity (*χ* ^2^ = 5.18 [*p* = .39], *I* ^2^ = 12.40%, *τ* ^2^ = 0.01). These findings indicate that bystander programs have a positive (beneficial) effect on rape myth acceptance 6 to 7 months post‐intervention. However, the small sample size in this meta‐analysis (*n* < 10) precluded ad hoc analysis of moderators or small study/publication bias.

#### 9.3.3 Gender attitudes

Six studies measured gender attitudes as an intervention outcome. These studies operationalized gender attitudes or gender‐role ideology through multiple‐item scales consisting of statements such as “If men pay for a date, they deserve something in return.”

All but two of these studies (Miller et al., 2014; Peterson et al., 2016) involved random assignment of participants to conditions. All but one study (Brokenshire, 2015) was published in a peer‐reviewed outlet; this study was an unpublished master's thesis. We collapsed gender attitude effect sizes into three timing intervals: (a) immediate posttest (i.e., 0 week to 1 week), (b) 2 to 4 months post‐intervention, and (c) 6 months to 1 year post‐intervention.

##### 9.3.3.1 Immediate post‐test effects

One study (Brokenshire, 2015) reported gender attitudes as an immediate posttest. This study indicated that bystander programs had a nonsignificant but negative effect on gender attitudes at immediate posttest (*g* = −0.41, 95% CI [−0.88, 0.06]).

##### 9.3.3.2 Two to four month intervention effects

**Figure 7:**
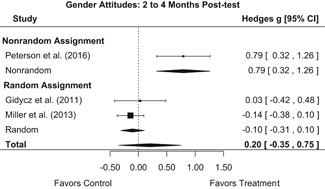
Forest plot of bystander intervention effects on gender attitudes—2 to 4 months post‐intervention

Three studies reported gender attitudes 2 to 4 months post‐intervention. As shown in the forest plot in Figure 7, the intervention effect for the one nonrandomized study was significant and positive (*g* = 0.79, 95% CI [0.32, 1.26]). The average effect for the two randomized studies was nonsignificant (*g* = −0.10, 95% CI [−0.35, 0.75]) with nonsignificant heterogeneity (*χ* ^2^ = 0.43 [*p* = .51], *I* ^2^ = 0.00%, *τ* ^2^ = 0). Across all studies (randomized and nonrandomized), the average intervention effect was nonsignificant (*g* = 0.20, 95% CI [−0.35, 0.75]) with significant between‐study heterogeneity (*χ* ^2^ = 12.02 [*p* = .00], *I* ^2^ = 84.17%, *τ* ^2^ = 0.20). Thus, these findings indicate that there is no evidence that bystander programs have an effect on gender attitudes 2 to 4 months post‐intervention. Again, however, the small sample size in this meta‐analysis (*n* < 10) precluded ad hoc analysis of moderators or small study/publication bias.

##### 9.3.3.3 Six month to one‐year intervention effects

**Figure 8:**
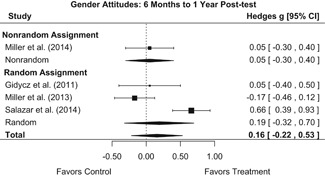
Forest plot of bystander intervention effects on gender attitudes—6 months to 1 year post‐intervention

Four studies reported gender attitudes 6 months to 1 year post‐intervention. As shown in the forest plot in Figure 8, the intervention effect for the one nonrandomized study was nonsignificant (*g* = 0.05, 95% CI [−0.30, 0.40]). The intervention effect for the three randomized studies was nonsignificant (*g* = 0.19, 95% CI [−0.32, 0.70]) with significant heterogeneity (*χ* ^2^ = 17.15 [*p* = .00], *I* ^2^ = 85.94%, *τ* ^2^ = 0.17). Across all studies (randomized and nonrandomized), the average intervention effect was nonsignificant (*g* = 0.16, 95% CI [−0.22, 0.53]) with significant heterogeneity (*χ* ^2^ = 18.00 [*p* = .00], *I* ^2^ = 80.07%, *τ* ^2^ = 0.12). Thus, there is no evidence that bystander programs had an effect on gender attitude outcomes 6 months to 1 year post‐intervention. The small sample size in this meta‐analysis (*n* < 10) precluded ad hoc analysis of moderators or small study/publication bias.

#### 9.3.4 Date rape attitudes

Four studies measured data rape attitudes. These studies operationalized date rape attitudes with multi‐item scales such as the College Date Rate Attitude Survey (Lanier & Elliot, 1997) and the Rape Attitudes and Beliefs Scale (Burgess, 2007). Such scales asked respondents to rate their approval of statements such as, “Many women pretend they don't want to have sex because they don't want to appear ‘easy.’” All four of these studies involved random assignment of participants to conditions. Two of these studies were published in peer‐reviewed outlets (Banyard et al., 2007; Salazar et al., 2014) and two were unpublished theses/dissertations (Brokenshire, 2015; Chiriboga, 2016). More than one study reported immediate posttest effects, so we synthesized these together. The timing of follow‐up waves was too disparate between studies to permit synthesis; thus, we report these results individually.

##### 9.3.4.1 Immediate post‐test effects

**Figure 9:**
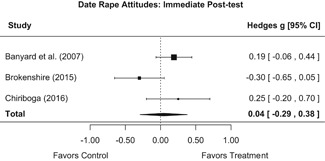
Forest plot of bystander intervention effects on date rape attitudes at immediate posttest

Three studies reported date rape attitudes as an immediate posttest outcome. As shown in Figure 9, there was no evidence that bystander interventions had an effect on date rape attitudes at immediate follow‐up (*g* = 0.04, 95% CI [−0.29, 0.38]; *χ* ^2^ = 5.68 [*p* = .06], *I* ^2^ = 64.81%, *τ* ^2^ = 0.06). The small sample size in this meta‐analysis (*n* < 10) precluded ad hoc analysis of moderators or small study/publication bias.

**Figure 10:**
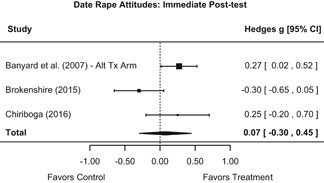
Forest plot of bystander intervention effects on date rape attitudes at immediate posttest—sensitivity analysis with alternative treatment arm for Banyard et al. (2007)

Sensitivity analysis in which we replaced the Banyard et al. (2007) one‐session intervention with the three‐session intervention did not substantively change these findings (*g* = 0.07, 95% CI [−0.30, 0.45]) and indicated significant between‐study heterogeneity in effects (*χ* ^2^ = 7.07 [*p* = .03], *I* ^2^ = 70.93%, *τ* ^2^ = 0.08). See Figure 10.

##### 9.3.4.2 Follow‐up effects

Two studies reported a date rape attitude outcome beyond the immediate posttest. The timing of these measures was too disparate to permit synthesis of effect sizes. Banyard et al. (2007) reported date rape attitudes 2 months post‐intervention for both the (focal) one‐session intervention (*g* = 0.23, 95% CI [−0.06, 0.52]) and the (alternative) three‐session intervention (*g* = 0.21, 95% CI [−0.06, 0.48]). Neither of these intervention effects was statistically significant. Salazar et al. (2014) reported date rape attitudes 6 months post‐intervention and reported a positive and statistically significant intervention effect (*g* = 0.91, 95% CI [0.64, 1.18]).

#### 9.3.5 Noticing a sexual assault or its warning signs

Four studies reported a measure of whether participants noticed a sexual assault occurring. Studies operationalized this outcome in a number of ways, including a single item developed by Burn (2009): “At a party or bar, I am probably too busy to be aware of whether someone is at risk for sexually assaulting someone.” All but one of these studies (Senn & Forrest, 2016) involved random assignment of participants to conditions and all but one (Brokenshire, 2015) were published in a peer‐reviewed outlet. This specific report was an unpublished master's thesis. We collapsed noticing sexual assault effect sizes into three timing intervals: (a) immediate posttest (i.e., 0 week to 1 week), (b) 1 month to 4 months, and (c) 1 year.

##### 9.3.5.1 Immediate post‐test effects

**Figure 11:**
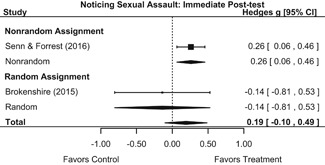
Forest plot of bystander intervention effects on noticing sexual assault —immediate posttest

Two studies reported noticing sexual assault as an immediate posttest outcome (i.e., 0 week to 1 week post intervention). As shown in the forest plot in Figure 11, the intervention effect for the one nonrandomized study was significant and positive (*g* = 0.26, 95% CI [0.06, 0.46]). Conversely, the effect for the one randomized study was nonsignificant (*g* = −0.14, 95% CI [−0.81, 0.53]). Across the two studies, the average intervention effect was nonsignificant (*g* = 0.19, 95% CI [−0.10, 0.45]) with nonsignificant heterogeneity (*χ* ^2^ = 1.27 [*p* = .26], *I* ^2^ = 21.50%, *τ* ^*2*^ = 0.02). Thus, at immediate post‐intervention, there was no evidence that bystander programs have an effect on participants' reports of noticing a sexual assault. The small sample size in this meta‐analysis (*n* < 10) precluded ad hoc analysis of moderators or small study/publication bias.

##### 9.3.5.2 One to four‐month follow‐up effects

**Figure 12:**
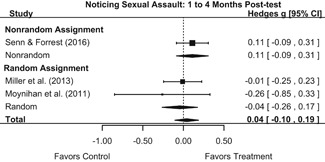
Forest plot of bystander intervention effects on noticing sexual assault—1 to 4 months post‐intervention

Three studies reported noticing sexual assault as an outcome 1 to 4‐month post‐intervention. As shown in the forest plot in Figure 12, the intervention effect for the one nonrandomized study was nonsignificant (*g* = 0.11, 95% CI [−0.09, 0.31]). The average effect for the two randomized studies was also nonsignificant (*g* = −0.04, 95% CI [−0.26, 0.17]) with nonsignificant heterogeneity (*χ* ^2^ = 0.60 [*p* = .44], *I* ^2^ = 0.00%, *τ* ^2^ = 0). Across all studies (randomized and nonrandomized), the average intervention effect was nonsignificant (*g* = 0.04, 95% CI [−0.10, 0.19]) with nonsignificant heterogeneity (*χ* ^2^ = 1.66 [*p* = .44], *I* ^2^ = 0.00%, *τ* ^2^ = 0). Thus, there was no evidence that bystander programs have an effect on participants' reports of noticing a sexual assault 1 to 4‐month post‐intervention. The small sample size in this meta‐analysis (*n* < 10) precluded ad hoc analysis of moderators or small study/publication bias.

##### 9.3.5.3 One year follow‐up effects

One study reported noticing sexual assault as an intervention outcome 1 year post‐intervention. The effect size from this study (Miller et al., 2013) was nonsignificant (*g* = −0.06, 95% CI [−0.13, 0.41]), thus providing no evidence that the program in this study affected respondents' noticing a sexual assault 1 year post‐intervention.

#### 9.3.6 Identifying a situation as appropriate for intervention

Six studies reported a measure of participants' identification of a situation as appropriate for intervention. Studies frequently operationalized this concept using some adaptation of Burn's (2009) Failure to Identify Situation as High Risk scale (e.g., “In a party or bar situation, I think I might be uncertain as to whether someone is at‐risk for being sexually assaulted”). All but three of these studies (Amar et al., 2015; Baker et al., 2014; Senn & Forrest, 2016) involved random assignment of participants to conditions and all but one (Addison, 2015) was published in a peer‐reviewed outlet. This specific report was an unpublished doctoral dissertation. We collapsed noticing sexual assault effect sizes into three timing intervals: (a) immediate posttest (i.e., 0 week to 1 week), (b) 1 month to 4 months post‐intervention, and (c) 6 months post‐intervention.

##### 9.3.6.1 Immediate post‐test effects

**Figure 13:**
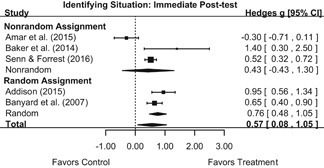
Forest plot of bystander intervention effects on identifying a situation as appropriate for intervention—immediate posttest

Five studies reported identification of a situation as appropriate for intervention as an immediate posttest outcome (i.e., 0 week to 1 week post‐intervention). As shown in the forest plot in Figure 13. The average intervention effect for the nonrandomized studies was nonsignificant (*g* = 0.43, 95% CI [−0.43, 1.30]) with significant heterogeneity across studies (*χ* ^2^ = 15.74 [*p* = .00], *I* ^2^ = 91.24%, *τ* ^2^ = 0.49). The average intervention effect for the randomized studies was significant and positive (*g* = 0.76, 95% CI [0.48, 1.05]), however, with nonsignificant heterogeneity (*χ* ^2^ = 1.58 [*p* = .21], *I* ^2^ = 36.78%, *τ* ^2^ = 0.02). Across all studies (randomized and nonrandomized), the average intervention effect was significant and positive (*g* = 0.57, 95% CI [0.08, 1.05]) with significant heterogeneity across studies (*χ* ^2^ = 23.31 [*p* = .00], *I* ^2^ = 89.71%, *τ* ^2^ = 0.25). Thus, at immediate post‐intervention, bystander programs have a positive (beneficial) and significant beneficial effect on participants' identification of a situation as appropriate for intervention. The small sample size in this meta‐analysis (*n* < 10) precluded ad hoc analysis of moderators or small study/publication bias.

**Figure 14:**
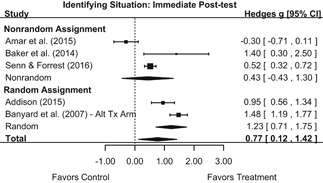
Forest plot of bystander intervention effects on identifying a situation as appropriate for intervention—immediate posttest with alternative Tx arm for Banyard et al. (2007)

We conducted a sensitivity analysis in which we replaced the Banyard et al. (2007) one‐session intervention arm with the three‐session intervention arm. As shown in Figure 14, findings were substantively similar to those from the main analysis. The intervention effect for the two randomized studies (which included Banyard et al.) was still significant and positive (*g* = 1.23, 95% CI [0.71, 1.75]; *χ* ^2^ = 4.49 [*p* = .03], *I* ^2^ = 77.75%, *τ* ^2^ = 0.011). The average intervention effect across all studies was also still significant and positive (*g* = 0.77, 95% CI [0.12, 1.42]; *χ* ^2^ = 56.05 [*p* = .00], *I* ^2^ = 93.82%, *τ* ^2^ = 0.47). Importantly, the replacement of Banyard et al.'s one‐session intervention arm with their three‐session intervention arm increased the magnitude of the average intervention effect for the randomized studies (0.76 to 1.23) as well as for the total sample (0.57 to 0.77).

##### 9.3.6.2 One to four‐month follow‐up effects

**Figure 15:**
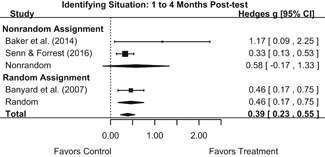
Forest plot of bystander intervention effects on identifying a situation as appropriate for intervention – 1‐ to 4‐month follow‐up

Three studies reported identification of a situation as appropriate for intervention 1‐ to 4‐month post‐intervention. The effect size for one of these studies (Senn & Forrest, 2016) fell more than two standard deviations below the mean of the distribution for the total sample (*g* = 0.26) so we Winsorized it by replacing it with the value that fell exactly two standard deviations below the mean (*g* = 0.33). As shown in the forest plot in Figure 15, the average intervention effect for the two nonrandomized studies was nonsignificant (*g* = 0.58, 95% CI [−0.17, 1.33]) with nonsignificant heterogeneity (*χ* ^2^ = 2.26 [*p* = .13], *I* ^2^ = 55.71%, *τ* ^2^ = 0.20). The intervention effect for the one randomized study was significant and positive (*g* = 0.46, 95% CI [0.17, 0.75]). Across all studies (randomized and nonrandomized), the average intervention effect was significant and positive (*g* = 0.39, 95% CI [0.23, 0.55]) with nonsignificant heterogeneity across studies (*χ* ^2^ = 2.59 [*p* = .27], *I* ^2^ = 0.01%, *τ* ^2^ = 0). Thus, the overall mean effect size indicates that bystander programs have a significant beneficial effect on participants' identification of a situation as appropriate for intervention 1‐ to 4‐month post‐intervention. The small sample size in this meta‐analysis (*n* < 10) precluded ad hoc analysis of moderators or small study/publication bias.

We conducted a sensitivity analysis to determine whether Winsorizing the effect size for Senn and Forrest (2016) affected our overall findings. Running the meta‐analysis with the original effect size for this study produced nearly identical findings to those from the main analysis for both the two nonrandomized studies (*g* = 0.55, 95% CI [−0.28, 1.39]) and the total sample containing both randomized and nonrandomized studies (*g* = 0.37, 95% CI [0.15, 0.59]). Tests for heterogeneity revealed nonsignificant heterogeneity, although specific values were slightly different than those from the main analysis for both the nonrandomized studies (*χ* ^2^ = 2.65 [*p* = .10], *I* ^2^ = 62.26%, *τ* ^2^ = 0.26) and the total sample (*χ* ^2^ = 3.56 [*p* = .17], *I* ^2^ = 26.48%, *τ* ^2^ = 0.01).

**Figure 16:**
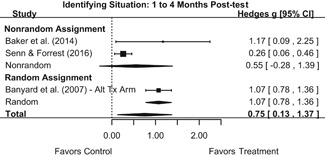
Forest plot of bystander intervention effects on identifying a situation as appropriate for intervention – 1‐ to 4‐month follow‐up with alternative treatment arm for Banyard et al. (2007)

We also conducted a sensitivity analysis in which we replaced the Banyard et al. (2007) one‐session intervention arm with the three‐session intervention arm. Inspection of the distribution of effect sizes revealed that there were no outliers in the total sample containing Banyard et al.'s three‐session intervention arm. As indicated in Figure 16, findings were substantively similar to those from the main analysis. The intervention effect for Banyard et al. (2007), the one randomized study in the sample, remained significant and positive (*g* = 1.07, 95% CI [0.78, 1.36]). The overall average effect for randomized and nonrandomized studies) also remained significant and positive (*g* = 0.75, 95% CI [0.13, 1.37]) with significant heterogeneity across studies (*χ* ^2^ = 21.60 [*p* = .00], *I* ^2^ = 88.54%, *τ* ^2^ = 0.23).

##### 9.3.6.3 Six month follow‐up effects

One study reported participants' identification of a situation as appropriate for intervention as an outcome 6 months post‐intervention. The effect size from this study (Salazar et al., 2014) was significant and positive (*g* = 1.56, 95% CI [1.25, 1.87]).

#### 9.3.7 Taking responsibility for acting/intervening

Four studies reported a measure of participants' taking responsibility for acting/intervening when witnessing violence or its warning signs. Studies operationalized this outcome using either the full or an adapted version of the Failure to Take Intervention Responsibility scale developed by Burn (2009), which consisted of items such as “I am less likely to intervene to reduce a person's risk of sexual assault if I think she/he made choices that increased their risk.” Two studies (Banyard et al., 2007; Moynihan, Banyard, Arnold, Eckstein, & Stapleton, 2011) involved random assignment of participants to conditions and two (Amar et al., 2015; Senn & Forrest, 2016) did not. All four studies were published in a peer‐reviewed outlet. We collapsed noticing sexual assault effect sizes into two timing intervals: (a) immediate posttest (i.e., 0 week to 1 week) and (b) 1 month to 4 months post‐intervention.

##### 9.3.7.1 Immediate post‐test effects

**Figure 17:**
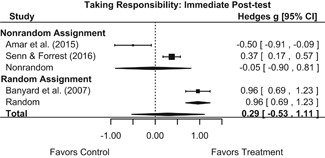
Forest plot of bystander intervention effects on taking responsibility to act/intervene – immediate posttest

Three studies reported taking responsibility for acting/intervening as an immediate posttest outcome (i.e., 0 week to 1 week post‐intervention). As shown in the forest plot in Figure 17, the intervention effect for the two nonrandomized studies was nonsignificant (*g* = −0.05, 95% CI [−0.90, 0.81]) with significant heterogeneity across studies (*χ* ^2^ = 14.00 [*p* = .00], *I* ^2^ = 92.85%, *τ* ^2^ = 0.35). The intervention effect for the randomized study was significant and positive (*g* = 0.96, 95% CI [0.69, 1.23]). The overall average intervention effect (for the randomized and nonrandomized studies) was nonsignificant (*g* = 0.29, 95% CI [−0.53, 1.11]) with significant heterogeneity (*χ* ^2^ = 34.30 [*p* = .00], *I* ^2^ = 96.07%, *τ* ^2^ = 0.50). The overall average intervention effect indicates that, at immediate post‐intervention, there is no evidence that bystander programs have an effect on participants' taking responsibility for acting/intervening when witnessing violence or its warning signs. The small sample size in this meta‐analysis (*n* < 10) precluded ad hoc analysis of moderators or small study/publication bias.

**Figure 18:**
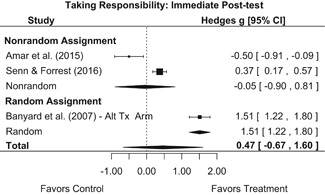
Forest plot of bystander intervention effects on taking responsibility to act/intervene – immediate posttest with alternative Tx arm for Banyard et al. (2007)

We conducted a sensitivity analysis in which we replaced the Banyard et al. (2007) one‐session intervention arm with the three‐session intervention arm. As indicated in Figure 18, findings were substantively similar to those from the main analysis. The intervention effect for Banyard et al. (2007), the single randomized study, was significant and positive (*g* = 1.51, 95% CI [1.22, 1.80]). The overall average intervention effect (for randomized and nonrandomized studies) was nonsignificant (*g* = 0.47, 95% CI [−0.67, 1.60]) with significant heterogeneity across studies (*χ* ^2^ = 69.20 [*p* = .00], *I* ^2^ = 97.83%, *τ* ^2^ = 0.98).

##### 9.3.7.2 One to four‐month follow‐up effects

**Figure 19:**
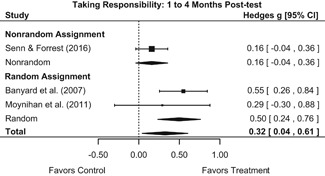
Forest plot of bystander intervention effects on taking responsibility to act/intervene – 1 to 4 months posttest

Three studies reported taking responsibility for acting/intervening 1‐ to 4‐month post‐intervention. As shown in the forest plot in Figure 19, the intervention effect for the one nonrandomized study was nonsignificant (*g* = 0.16, 95% CI [−0.04, 0.36]). The average intervention effect for the two randomized studies was significant and positive (*g* = 0.50, 95% CI [0.24, 0.76]) with nonsignificant heterogeneity (*χ* ^2^ = 0.60 [*p* = .44], *I* ^2^ = 0.00%, *τ* ^2^ = 0). Overall, the average intervention effect (for the randomized and nonrandomized studies) was significant and positive (*g* = 0.32, 95% CI [0.04, 0.61]) with nonsignificant heterogeneity (*χ* ^2^ = 4.68 [*p* = .10], *I* ^2^ = 56.86%, *τ* ^2^ = 0.03). Thus, the results indicate that bystander programs have a significant positive (beneficial) effect on participants' taking responsibility for acting/intervening when witnessing violence or its warning signs 1‐ to 4‐month post‐intervention. The small sample size in this meta‐analysis (*n* < 10) precluded ad hoc analysis of moderators or small study/publication bias.

**Figure 20:**
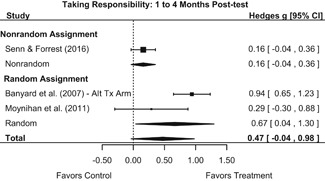
Forest plot of bystander intervention effects on taking responsibility to act/intervene – 1‐ to 4‐month posttest with alternative Tx arm for Banyard et al. (2007)

We conducted a sensitivity analysis in which we replaced the Banyard et al. (2007) one‐session intervention arm with the three‐session intervention arm. As indicated in Figure 20, substituting this intervention arm resulted in substantively different findings from those in the main analysis. The intervention effect for the two randomized studies (which included Banyard et al., 2007) remained significant and positive (*g* = 0.67, 95% CI [0.04, 1.30]) with nonsignificant heterogeneity (*χ* ^2^ = 3.76 [*p* = .05], *I* ^2^ = 73.37%, *τ* ^2^ = 0.16). However, the overall average intervention effect (for randomized and nonrandomized studies) was nonsignificant (*g* = 0.47, 95% CI [−0.04, 0.98]) with significant heterogeneity across studies (*χ* ^2^ = 18.84 [*p* = .00], *I* ^2^ = 86.59%, *τ* ^2^ = 0.17).

#### 9.3.8 Knowing strategies for helping/intervening

Four studies reported a measure of participants' knowledge of strategies for helping/intervening. Studies operationalized this concept using Burn's (2009) two‐item Failure to Intervene Due to Skills Deficit scale, which asked respondents to rate their agreement with statements such as “Although I would like to intervene when a guy's sexual conduct is questionable, I am not sure I would know what to say or do.” Two studies (Brokenshire, 2015; Potter, Stapleton, & Moynihan, 2008) involved random assignment of participants to conditions and two (Amar et al., 2015, Senn & Forrest, 2016) did not. All but one study (Brokenshire, 2015) was published in a peer‐reviewed outlet. This specific report was an unpublished master's thesis. We collapsed knowing strategies for helping/intervening into two timing intervals: (a) immediate posttest (i.e., 0 week to 2 weeks) and (b) 4 months post‐intervention.

##### 9.3.8.1 Immediate post‐test effects

**Figure 21:**
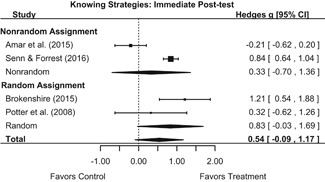
Forest plot of bystander intervention effects on knowing strategies for helping/intervening‐ immediate posttest

Four studies reported knowing strategies for helping/intervening as an immediate posttest outcome (i.e., 0 week to 2 weeks post‐intervention). As shown in the forest plot in Figure 21, the average intervention effect for the two nonrandomized studies was nonsignificant (*g* = 0.33, 95% CI [−0.70, 1.36]) with significant heterogeneity across studies (*χ* ^2^ = 20.38 [*p* = .00], *I* ^2^ = 95.10%, *τ* ^2^ = 0.52). The treatment effect for the randomized studies was nonsignificant (*g* = 0.83, 95% CI [−0.03, 1.69]) with nonsignificant heterogeneity (*χ* ^2^ = 2.29 [*p* = .13], *I* ^2^ = 56.32%, *τ* ^2^ = 0.22). Overall, the average intervention effect (for the randomized and nonrandomized studies) was nonsignificant (*g* = 0.54, 95% CI [−0.09, 1.17]) with significant heterogeneity (*χ* ^2^ = 23.50 [*p* = .00], *I* ^2^ = 86.46%, *τ* ^2^ = 0.33). Thus, at immediate post‐intervention, there is no evidence that bystander programs have an effect on participants' knowledge of strategies for helping/intervening. The small sample size in this meta‐analysis (*n* < 10) precluded ad hoc analysis of moderators or small study/publication bias.

##### 9.3.8.2 Four‐month follow‐up effects

One study (Senn & Forrest, 2016) reported knowing strategies for helping/intervening 4 months post‐intervention. The effect size for this study was significant and positive (*g* = 0.63, 95% CI [0.43, 0.83]).

#### 9.3.9 Bystander efficacy

Eleven studies reported a measure of bystander efficacy as an outcome. These studies operationalized bystander efficacy using a modified or adapted version of the Bystander Efficacy Scale (Banyard et al., 2007). This scale asked participants to rate their confidence in their ability to perform behaviors such as “try and stop or discourage someone who is spreading rumors online about another person's body or sexual behavior” or “do something to help a very drunk person who is being brought upstairs to a bedroom by a group of people at a party.” All but three of these studies (Baker et al., 2014; Peterson et al., 2016; Senn & Forrest, 2016) involved random assignment of participants to conditions and all but one (Addison, 2015) was published in a peer‐reviewed outlet. This specific report was an unpublished dissertation. We collapsed bystander efficacy effect sizes into three timing intervals: (a) immediate posttest (i.e., 0 week to 1 week), (b) 1 month to 4 months post‐intervention, and (3) 6 months post‐intervention.

##### 9.3.9.1 Immediate post‐test effects

**Figure 22:**
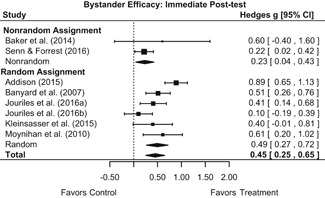
Forest plot of bystander intervention effects on bystander efficacy – immediate posttest

Eight studies reported bystander efficacy as an immediate posttest outcome (i.e., 0 week to 1 week post‐intervention). As shown in the forest plot in Figure 22, the average intervention effect for the two nonrandomized studies was significant and positive (*g* = 0.23, 95% CI [0.04, 0.43]) with nonsignificant heterogeneity (*χ* ^2^ = 0.53 [*p* = .46], *I* ^2^ = 0.00%, *τ* ^2^ = 0). The average intervention effect for the six randomized studies was significant and positive (*g* = 0.49, 95% CI [0.27, 0.72]) with significant heterogeneity across individual studies (*χ* ^2^ = 18.48 [*p* = .00], *I* ^2^ = 70.51%, *τ* ^2^ = 0.05). Across all studies (randomized and nonrandomized), the average intervention effect was significant and positive (*g* = 0.45, 95% CI [0.25, 0.65]) with significant heterogeneity across studies (*χ* ^2^ = 25.17 [*p* = .00], *I* ^2^ = 69.78%, *τ* ^2^ = 0.05). Thus, at immediate post‐intervention, bystander programs had a significant and positive effect on bystander efficacy. The small sample size in this meta‐analysis (*n* < 10) precluded ad hoc analysis of moderators or small study/publication bias.

**Figure 23:**
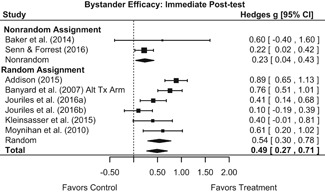
Forest plot of bystander intervention effects on bystander efficacy – immediate posttest with alternative Tx arm for Banyard et al. (2007)

We conducted a sensitivity analysis in which we replaced the Banyard et al. (2007) one‐session intervention arm with the three‐session intervention arm. As indicated in Figure 23, findings were substantively similar to those from the main analysis. The average intervention effect for the six randomized studies (which included Banyard et al.) was significant and positive (*g* = 0.54, 95% CI [0.30, 0.78]) with significant heterogeneity (*χ* ^2^ = 21.05 [*p* = .00], *I* ^2^ = 74.41%, *τ* ^2^ = 0.07). The overall average intervention effect (for randomized and nonrandomized studies) was also significant and positive (*g* = 0.49, 95% CI [0.27, 0.71]) with significant heterogeneity across studies (*χ* ^2^ = 30.32 [*p* = .00], *I* ^2^ = 74.30%, *τ* ^2^ = 0.07).

##### 9.3.9.2 One to four‐month follow‐up effects

**Figure 24:**
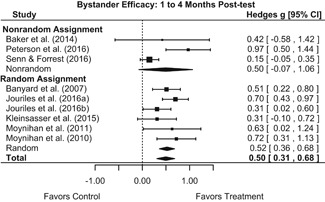
Forest plot of bystander intervention effects on bystander efficacy – 1‐ to 4‐month posttest

Nine studies reported bystander efficacy 1 to 4 months post‐intervention. As shown in Figure 24, the average intervention effect for the three nonrandomized studies was nonsignificant (*g* = 0.50, 95% CI [−0.07, 1.06]) with significant heterogeneity (*χ* ^2^ = 10.03 [*p* = .01], *I* ^2^ = 76.18%, *τ* ^2^ = 0.18). The average intervention effect for the six randomized studies was significant and positive (*g* = 0.52, 95% CI [0.36, 0.68]) with nonsignificant heterogeneity (*χ* ^2^ = 5.65 [*p* = .34], *I* ^2^ = 20.50%, *τ* ^2^ = 0.01). Across all studies (randomized and nonrandomized), the average intervention effect was significant and positive (*g* = 0.50, 95% CI [0.31, 0.68]) but with significant heterogeneity (*χ* ^2^ = 20.19 [*p* = .01], *I* ^2^ = 58.56%, *τ* ^2^ = 0.04). Thus, bystander programs have a significant positive (beneficial) effect on bystander efficacy at 1‐ to 4‐month post‐intervention. The small sample size in this meta‐analysis (*n* < 10) precluded ad hoc analysis of moderators or small study/publication bias.

**Figure 25:**
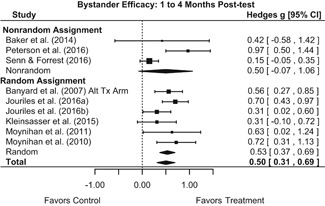
Forest plot of bystander intervention effects on bystander efficacy – 1‐ to 4‐month posttest with alternative Tx arm for Banyard et al. (2007)

We conducted a sensitivity analysis in which we replaced the Banyard et al. (2007) one‐session intervention arm with the three‐session intervention arm. As indicated in Figure 25, findings were substantively similar to those from the main analysis. The average intervention effect for the six randomized studies (which included Banyard et al.) was significant and positive (*g* = 0.53, 95% CI [0.37, 0.69]) with nonsignificant heterogeneity (*χ* ^2^ = 5.68 [*p* = .34], *I* ^2^ = 20.90%, *τ* ^2^ = 0.01). Across all studies (randomized and nonrandomized), the average intervention effect was also significant and positive (*g* = 0.50, 95% CI [0.31, 0.69]) but with significant heterogeneity across studies (*χ* ^2^ = 20.66 [*p* = .01], *I* ^2^ = 59.10%, *τ* ^2^ = 0.04).

##### 9.3.9.3 Six month follow‐up effects

One study (Salazar et al., 2014) reported bystander efficacy as an outcome 6 months post‐intervention. Since this study measured bystander efficacy 2 to 6 months later than all other studies reporting this outcome, we elected to report it separately. Findings from this particular study indicated a nonsignificant program effect (*g* = 0.13, 95% CI [−0.14, 0.40]) 6 months post‐intervention.

#### 9.3.10 Bystander intentions

Eleven studies reported a measure of bystander intentions as an outcome. Studies operationalized this outcome using the full or adapted Bystander Intent to Help Scale (Banyard, Moynihan, Cares, & Warner, 2014), which asks participants their likelihood to engage in bystander behavior (e.g., “I stop and check in on someone who looks intoxicated when they are being taken upstairs at a party.”). All but four of these studies (Amar et al., 2015; Miller et al., 2014; Peterson et al., 2016; Senn & Forrest, 2016) involved random assignment of participants to conditions and all but one (Brokenshire, 2015) was published in a peer‐reviewed outlet. This specific report was an unpublished master's thesis. We collapsed bystander efficacy effect sizes into three timing intervals: (a) immediate posttest (i.e., 0 week to 1 week), (b) 1 month to 4 months post‐intervention, and (c) 6 months to 1 year post‐intervention.

##### 9.3.10.1 Immediate post‐test effects

**Figure 26:**
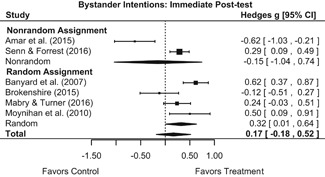
Forest plot of bystander intervention effects on bystander intentions – immediate posttest

Six studies reported bystander intentions as an immediate posttest outcome (i.e., 0 week to 1 week post‐intervention). As shown in the forest plot in Figure 26, the average intervention effect for the two nonrandomized studies was nonsignificant (*g* = −.15, 95% CI [−1.04, 0.74]) but with significant heterogeneity across studies (*χ* ^2^ = 15.31 [*p* = .00], *I* ^2^ = 93.47%, *τ* ^2^ = 0.39). The average intervention effect for the four randomized studies was significant and positive (*g* = 0.32, 95% CI [0.01, 0.64]) with significant heterogeneity (*χ* ^2^ = 10.94 [*p* = .01], *I* ^2^ = 73.17%, *τ* ^2^ = 0.07). Across all studies (randomized and nonrandomized), the average intervention effect was nonsignificant (*g* = 0.17, 95% CI [−0.18, 0.52]) but with significant heterogeneity (*χ* ^2^ = 30.25 [*p* = .00], *I* ^2^ = 87.57%, *τ* ^2^ = 0.16). The small sample size in this meta‐analysis (*n* < 10) precluded ad hoc analysis of moderators or small study/publication bias.

Interestingly, the average intervention effect for the randomized studies was significant and positive, but when synthesized with the two nonrandomized studies, the average intervention effect was nonsignificant. This attenuation appears to be driven by the extreme (negative) effect size reported by Amar et al. (2015). However, preliminary inspection of the distribution of effect sizes indicated this was not an outlier; thus, we did not Winsorize it in our main analysis. Nevertheless, in light of these finding we ran a sensitivity analysis in which we dropped Amar et al. (2015) from the sample. When removing Amar et al. (2015), the overall average effect size was significant and positive (*g* = 0.32, 95% CI [0.08, 0.56]) with significant heterogeneity (*χ* ^2^ = 11.26 [*p* = .02], *I* ^2^ = 67.19%, *τ* ^2^ = 0.04).

**Figure 27:**
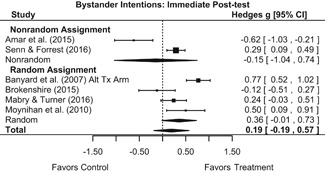
Forest plot of bystander intervention effects on bystander intentions – immediate posttest with alternative Tx arm for Banyard et al. (2007)

We conducted a sensitivity analysis in which we replaced the Banyard et al. (2007) one‐session intervention arm with the three‐session intervention arm. As indicated in Figure 27, findings were substantively similar to those from the main analysis. The average intervention effect for the six randomized studies (which included Banyard et al.) was nonsignificant (*g* = 0.36, 95% CI [−0.01, 0.73]) with significant heterogeneity across studies (*χ* ^2^ = 16.34 [*p* = .00], *I* ^2^ = 81.16%, *τ* ^2^ = 0.12). Across all studies (randomized and nonrandomized), the average intervention effect was nonsignificant (*g* = 0.19, 95% CI [−0.19, 0.57]) but with significant heterogeneity across individual study effect sizes (*χ* ^2^ = 37.74 [*p* = .00], *I* ^2^ = 89.59%, *τ* ^2^ = 0.20).

##### 9.3.10.2 One to four‐month follow‐up effects

**Figure 28:**
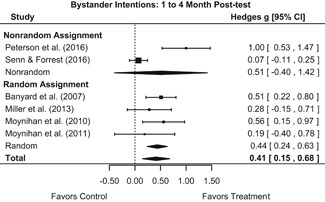
Forest plot of bystander intervention effects on bystander intentions – 1 to 4 months post‐intervention

Six studies reported a measure of bystander intentions 1 to 4 months post‐intervention. As shown in Figure 28, the average intervention effect for the nonrandomized studies was nonsignificant (*g* = 0.51, 95% CI [−0.40, 1.42) with significant heterogeneity across individual effect sizes (*χ* ^2^ = 13.16 [*p* = .00], *I* ^2^ = 92.40%, *τ* ^2^ = 0.40). The average intervention effect for the randomized studies was significant and positive (*g* = 0.44, 95% CI [0.24, 0.63]) with nonsignificant heterogeneity (*χ* ^2^ = 1.77 [*p* = .62], *I* ^2^ = 0.00%, *τ* ^2^ = 0). Across all studies (randomized and nonrandomized), the average intervention effect was significant and positive (*g* = 0.41, 95% CI [0.15, 0.68]) but with significant heterogeneity across studies (*χ* ^2^ = 18.64 [*p* = .00], *I* ^2^ = 70.16%, *τ* ^2^ = 0.07). Overall, these findings indicate bystander programs have a significant positive (beneficial) effect on bystander intentions 1‐ to 4‐month post‐intervention. The small sample size in this meta‐analysis (*n* < 10) precluded ad hoc analysis of moderators or small study/publication bias.

**Figure 29:**
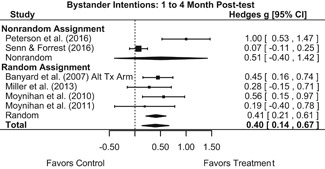
Forest plot of bystander intervention effects on bystander intentions – 1 to 4 months post‐intervention with alternative Tx arm for Banyard et al. (2007)

We conducted a sensitivity analysis in which we replaced the Banyard et al. (2007) one‐session intervention arm with the three‐session treatment arm. As indicated in Figure 29, findings were substantively similar to those from the main analysis. The average intervention effect for the four randomized studies (which included Banyard et al.) was significant and positive (*g* = 0.41, 95% CI [0.21, 0.61]) with nonsignificant heterogeneity effect sizes (*χ* ^2^ = 1.47 [*p* = .69], *I* ^2^ = 0.00%, *τ* ^2^ = 0). Across all studies, the average intervention effect was significant and positive (*g* = 0.40, 95% CI [0.14, 0.67]) with significant heterogeneity (*χ* ^2^ = 17.60 [*p* = .00], *I* ^2^ = 69.26%, *τ* ^2^ = 0.07).

##### 9.3.10.3 Six month to one year follow‐up effects

**Figure 30:**
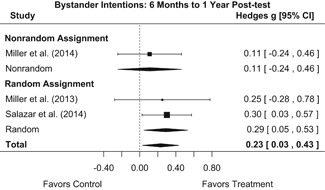
Forest plot of bystander intervention effects on bystander intentions – 6 months to 1 year post‐intervention

Three studies reported bystander intentions 6 months to 1 year post‐intervention. As shown in Figure 30, the program effect for the one nonrandomized study was nonsignificant (*g* = 0.11, 95% CI [−0.24, 0.46). The average intervention effect for the two randomized studies was significant and positive (*g* = 0.29, 95% CI [0.05, 0.53]) with nonsignificant heterogeneity across individual effect sizes (*χ* ^2^ = 0.03 [*p* = .87], *I* ^2^ = 0.00%, *τ* ^2^ = 0). Across all studies (randomized and nonrandomized), the average intervention effect was significant and positive (*g* = 0.23, 95% CI [0.03, 0.43]) with nonsignificant heterogeneity (*χ* ^2^ = 0.70 [*p* = .70], *I* ^2^ = 0.00%, *τ* ^2^ = 0). Overall, these findings indicate bystander programs had a significant positive (beneficial) effect on bystander intentions 6 months to 1 year post‐intervention.

#### 9.3.11 Bystander intervention

Thirteen studies reported a measure of bystander intervention as an outcome. All studies used a form of the Bystander Behaviors Scale (Banyard et al., 2007), which asked participants to indicate the extent to which they have engaged in bystander behavior (e.g., “walked a friend home from a party who has had too much to drink”). All but three of these studies (Miller et al., 2014; Peterson et al., 2016; Senn & Forrest, 2016) involved random assignment of participants to conditions and all but one (Jouriles et al., n.d.) was published in a peer‐reviewed outlet. This specific report was a draft of a manuscript under review for publication at the time of this review.

No studies measured bystander intervention at immediate post‐intervention. We thus collapsed bystander intervention effect sizes into two timing intervals: (a) 1 to 4 months post‐intervention and (b) 6 months to 1 year post‐intervention.

##### 9.3.11.1 One to four‐month follow‐up effects

**Figure 31:**
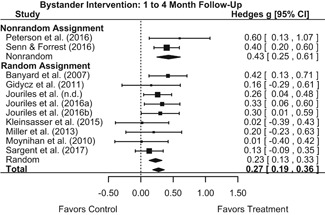
Forest plot of bystander intervention effects on bystander intervention – 1 to 4 months post‐intervention

Eleven studies reported bystander behavior 1‐ to 4‐month post‐intervention. Inspection of the distribution of effect sizes revealed that the effect size of one of the nonrandomized studies (Peterson et al., 2016) fell more than two standard deviations above the mean of the distribution. We therefore Winsorized this effect size (*g* = 0.63) by replacing it with the value that fell exactly two standard deviations above the mean (*g* = 0.60). As depicted in the forest plot in Figure 31 the average intervention effect for the two nonrandomized studies was significant and positive (*g* = 0.43, 95% CI [0.25, 0.61]) with nonsignificant heterogeneity (*χ* ^2^ = 0.59 [*p* = .44], *I* ^2^ = 0.00%, *τ* ^2^ = 0). The average intervention effect for the nine randomized studies was also significant and positive (*g* = 0.23, 95% CI [0.13, 0.33]) with nonsignificant heterogeneity (*χ* ^2^ = 5.44 [*p* = .71], *I* ^2^ = 0.00%, *τ* ^2^ = 0). Across all studies (randomized and nonrandomized), the average intervention effect was significant and positive (*g* = 0.27, 95% CI [0.19, 0.36]) with nonsignificant heterogeneity (*χ* ^2^ = 9.70 [*p* = .47], *I* ^2^ = 2.16%, *τ* ^2^ = 0). These findings indicate that, at 1‐ to 4‐month post‐intervention bystander programs have a significant positive (beneficial) effect on bystander intervention.

We conducted mixed‐effects meta‐regression to examine whether the effect of bystander programs on bystander intervention behavior varied based on the following: (a) gendered content of the program (i.e., sex of perpetrators and victims); (b) mixed‐ or single‐sex group implementation; (c) gender composition of the sample (i.e., proportion males); (d) education level of the sample (i.e., secondary school or college students); and (e) mean age of the sample. Although we had also planned to examine proportion of fraternity/sorority members in the sample and proportion of athletic team members as potential effect size moderators, these measures were rarely or never reported in the included studies and thus we were unable to examine them. Finally, due to the small number of included studies in this synthesis, we conducted separate bivariate regressions for each potential moderator, meaning that we estimated separate meta‐regression models for each potential moderator. As summarized in Table 4, none of these moderators exhibited a statistically significant bivariate association with effect size magnitude. The results from these moderator analyses should be interpreted cautiously, however, given the small number of included studies and our inability to estimate more comprehensive, multivariable meta‐regression models.

**Table 4:** Unstandardized bivariate meta‐regression coefficients for potential moderators of program effects on bystander intervention behavior – 1 to 4 months post‐intervention

Moderator	B	SE	95% CI
Sex of perpetrators & victims (gender neutral)	0.10	0.17	[−0.23, 0.43]
Mixed sex implementation (Yes)	0.13	0.17	[−0.21, 0.46]
Proportion males in sample	−0.00	0.00	[−0.01, 0.00]
Education level (secondary school)	−0.16	0.11	[−0.38, 0.05]
Age	0.04	0.02	[−0.01, 0.08]

*Notes*: The reference group for sex of perpetrators and victims is male perpetrators/female victims. The reference group for education level is college. Coefficients were estimated from separate bivariate regressions for each moderator.

**Figure 32:**
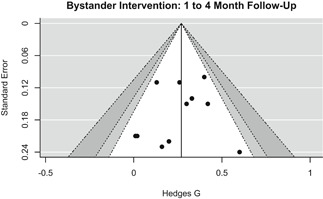
Contour enhanced funnel plot of bystander intervention 1 to 4 months post‐intervention

To examine small study and publication bias we created a contour‐enhanced funnel plot of the 11 effect sizes plotted against their standard errors (see Figure 32). Visual inspection of the funnel plot reveals an absence of adverse intervention effects. Given the absence of negative effects in the regions of statistical significance and nonsignificance, the results from this contour‐enhanced funnel plot indicate a potential risk of publication bias.

To further investigate the possibility of bias, we conducted an Egger test for funnel plot asymmetry. The results provided no significant evidence of small study effects (bias coefficient: 0.36; *t*: −0.60, *p* = .56). Finally, we also conducted a trim and fill analysis. With one study trimmed and filled, the resulting mean effect size was similar to that observed in the main analysis (trim‐and‐fill mean *g* = 0.28, 95% CI [0.20, 0.37]). With these collective findings, we therefore conclude that the meta‐analysis results shown in Figure 31 are likely robust to any small study/publication bias.

We also conducted meta‐regression to examine whether the effect of bystander programs on bystander intervention outcomes varied based on the following characteristics: (a) attrition at first follow‐up (i.e., 1 to 4 months post‐intervention), (b) random sequence ROB, (c) blinding of participants ROB, (d) incomplete data ROB, and (e) selective reporting ROB. We had also planned to examine blinding of assessment ROB, but the lack of variation in this measure precluded such analysis (i.e., all studies in this meta‐analysis were coded as high ROB for blinding of assessment). Due to the small sample size, we conducted bivariate regressions for each potential moderator.

As shown in Table 5, there was no evidence that attrition, blinding of participants, incomplete data, or selective reporting risk of bias assessments were associated with effect size magnitude. There was, however, evidence that studies rated as unclear risk of bias due to random sequence generation reported significantly smaller effect sizes than those at low risk of bias. There was no evidence that studies rated as high risk of bias due to random sequence generation reported significantly different effect sizes from those at low risk of bias.

**Table 5:** Unstandardized bivariate meta‐regression coefficients for attrition and risk of bias moderators of the relationship between bystander programs and bystander intervention 1‐ to 4‐month post‐Tx

Moderator	B	SE	95% CI
Attrition at first follow‐up	−0.39	0.38	[−1.14, 0.35]
Random sequence bias			
High	0.13	0.11	[−0.09, 0.35]
Unclear	−0.21*	0.10	[−0.41, −0.00]
Blinding of participants			
Unclear	−0.16	0.11	[−0.37, 0.06]
Incomplete data			
High	0.20^+^	0.11	[−0.00, 0.41]
Unclear	−0.03	0.23	[−0.47, 0.41]
Selective reporting			
High	−0.12	0.24	[−0.58, 0.35]

*Notes*: *p < .05; ^+^ *p* < .10. Low is the reference group for all risk of bias predictors. For blinding of participants no studies were coded as high risk. For selective reporting no studies were coded as unclear risk. Coefficients were estimated from separate bivariate regressions for each moderator.

We conducted a sensitivity analysis to determine whether Winsorizing the effect size for Peterson et al. (2016) affected our overall findings. Running the meta‐analysis with the original effect size for this study produced identical findings to those from the main analysis for both the two non‐randomized studies (*g* = 0.43, 95% CI [0.25, 0.61]) and the total sample containing both randomized and non‐randomized studies (*g* = 0.27, 95% CI [0.19, 0.36]). Tests for heterogeneity revealed non‐significant heterogeneity, although specific values were slightly different than those from the main analysis for both the nonrandomized studies (*χ* ^2^ = 0.72 [*p* = .40], *I* ^2^ = 0.00%, *τ* ^2^ = 0) and the total sample (*χ* ^2^ = 9.93 [*p* = .45], *I* ^2^ = 2.63%, *τ* ^2^ = 0).

**Figure 33:**
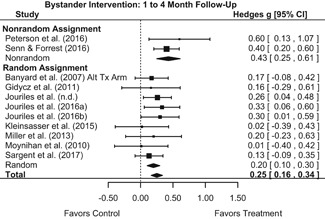
Forest plot of bystander intervention effects on bystander intervention – 1 to 4 months post‐intervention with alternative Tx arm for Banyard et al. (2007)

We also conducted two sensitivity analyses in which we replaced (a) the Banyard et al. (2007) one‐session intervention arm with the three‐session treatment arm and (b) the Jouriles et al. (n.d.) independent intervention arm with the monitored intervention arm (i.e., completed in a lab with supervision). As indicated in Figure 33, findings from the Banyard et al. sensitivity analysis were substantively similar to those from the main analysis. The average intervention effect for the nine randomized studies (which included Banyard et al.) was significant and positive (*g* = 0.20, 95% CI [0.10, 0.30]) with non‐significant heterogeneity (*χ* ^2^ = 3.64 [*p* = .89], *I* ^2^ = 0.00%, *τ* ^2^ = 0). The average intervention effect (for randomized and nonrandomized studies) was also significant and positive (*g* = 0.25, 95% CI [0.16, 0.34]) with nonsignificant heterogeneity (*χ* ^2^ = 9.10 [*p* = .52], *I* ^2^ = 0.93%, *τ* ^2^ = 0).

**Figure 34:**
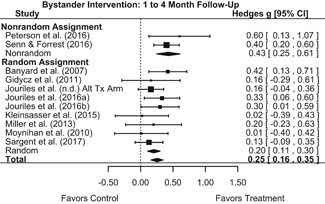
Forest plot of bystander intervention effects on bystander intervention – 1 to 4 months post‐intervention with alternative Tx arm for Jouriles et al. (n.d.)

Similarly, as indicated in Figure 34, findings from the Jouriles et al. (n.d.) sensitivity analysis were substantively similar to those from the main analysis. The average intervention effect for the nine randomized studies (which included Jouriles et al. (n.d.)) was significant and positive (*g* = 0.20, 95% CI [0.11, 0.30]) with nonsignificant heterogeneity (*χ* ^2^ = 5.60 [*p* = .69], *I* ^2^ = 0.00%, *τ* ^2^ = 0). The overall average effect (for randomized and nonrandomized studies) was also significant and positive (*g* = 0.25, 95% CI [0.16, 0.35]) with nonsignificant heterogeneity across individual study effect sizes (*χ* ^2^ = 10.80 [*p* = .37], *I* ^2^ = 12.69%, *τ* ^2^ = 0).

##### 9.3.11.2 Six month to one‐year follow‐up effects

**Figure 35:**
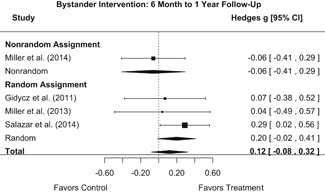
Forest plot of bystander intervention effects on bystander intervention ‐ 6 months to 1 year post‐intervention

Four studies reported bystander behavior 6 months to 1 year post‐intervention. As shown in the forest plot in Figure 35, the program effect for the single nonrandomized study in this subsample was nonsignificant (*g* = −0.06, 95% CI [−0.41, 0.29]) and the average intervention effect for the three randomized studies was nonsignificant (*g* = 0.20, 95% CI [−0.08, 0.32]) with nonsignificant heterogeneity (*χ* ^2^ = 1.08 [*p* = .58], *I* ^2^ = 0.00%, *τ* ^2^ = 0). Across all studies (randomized and nonrandomized), the average intervention effect was also nonsignificant (*g* = 0.12, 95% CI [−0.08, 0.32]) with nonsignificant heterogeneity (*χ* ^2^ = 2.60 [*p* = .46], *I* ^2^ = 14.23%, *τ* ^2^ = .01). Thus, these findings indicate that, at 6 months to 1 year post‐intervention, bystander programs have a significant positive (beneficial) effect on bystander intervention. The small sample size in this meta‐analysis (*n* < 10) precluded ad hoc analysis of moderators or small study/publication bias.

#### 9.3.12 Sexual assault perpetration

Six studies reported sexual assault perpetration as an outcome, asking participants to self‐report their perpetration of sexual coercion or sexual abuse. All but one of these studies (Miller et al., 2014) involved random assignment of participants to conditions. All six studies were published in a peer‐reviewed outlet.

No studies measured sexual assault perpetration at immediate post‐intervention. We thus collapsed bystander intervention effect sizes into two timing intervals: (a) 3 to 4 months post‐intervention and (2) 6 months to 1 year post‐intervention.

##### 9.3.12.1 Three to four‐month follow‐up effects

**Figure 36:**
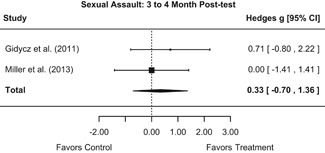
Forest plot of bystander intervention effects on sexual assault perpetration – 3 to 4 months post‐intervention

Two studies measured sexual assault perpetration 3 or 4 months post‐intervention. As shown in the forest plot in Figure 36, the average intervention effect across these two studies was nonsignificant (*g* = 0.33, 95% CI [−0.70, 1.36]) with nonsignificant heterogeneity across individual study effect sizes (*χ* ^2^ = 0.45 [*p* = .50], *I* ^2^ = 0.00%, *τ* ^2^ = 0). These findings provide no evidence that bystander programs have an effect on sexual assault perpetration at 3‐ to 4‐months post‐intervention.

##### 9.3.12.2 Six month to one‐year follow‐up effects

**Figure 37:**
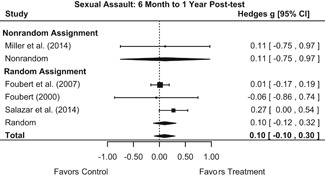
Forest plot of bystander intervention effects on sexual assault perpetration – 6 months to 1‐year post‐intervention

Four studies measured sexual assault perpetration 6 months to 1 year post‐intervention. As shown in the forest plot in Figure 37, the program effect for the one nonrandomized study was nonsignificant (*g* = 0.11, 95% CI [−0.75, 0.97]). The average intervention effect across the three randomized studies was nonsignificant (*g* = 0.10, 95% CI [−0.12, 0.32]) with nonsignificant heterogeneity (*χ* ^2^ = 2.56 [*p* = .28], *I* ^2^ = 35.66%, *τ* ^2^ = 0.01). Across all studies (randomized and nonrandomized), the average intervention was nonsignificant (*g* = 0.10, 95% CI [−0.10, 0.30]) with nonsignificant heterogeneity (*χ* ^2^ = 2.57 [*p* = .46], *I* ^2^ = 23.85%, *τ* ^2^ = 0.01). These findings provide no evidence that bystander programs have an effect on sexual assault perpetration 6 months to 1 year post‐intervention. The small sample size in this meta‐analysis (*n* < 10) precluded ad hoc analysis of moderators or small study/publication bias.

## 10 DISCUSSION

### 10.1 Summary of main results

#### 10.1.1 Objective 1: Bystander intervention effects on knowledge, attitudes, and behavior

##### 10.1.1.1 Knowledge/attitudes

Bystander intervention effects on knowledge/attitude outcomes varied widely across constructs. The most pronounced effect in this domain was on rape myth acceptance. The average program effect for this outcome was immediate and sustained across all reported follow‐up waves (i.e., from immediate posttest to 6‐ to 7‐month post‐intervention). At each of these follow‐up waves rape myth acceptance scores were approximately two‐fifths of a standard deviation lower for young people who participated in a bystander program relative to scores among the comparison group. To put this in context, one study in our meta‐analytic sample (Salazar et al., 2014) reported a preintervention mean score of 36.69 (*SD* = 10.29) on the Rape Myth Acceptance Scale, with a total possible score of 85 and higher scores indicating greater rape myth endorsement. Considering that the average effect size for rape myth acceptance at immediate posttest in our sample was 0.37, extrapolation suggests that participating in a bystander program decreased the mean rape myth acceptance score from 36.69 to 32.88 (i.e., (36.69 − [0.37 × 10.29]) = 32.88), a desirable change of approximately 4 points on an 85‐point scale.

Program effects on bystander efficacy were also fairly pronounced, with a significant effect observed at both immediate posttest and 1‐ to 4‐month post‐intervention. At both of these follow‐up waves measures of bystander efficacy were approximately one‐half of a standard deviation higher for bystander program participants than for the comparison group. Again, for illustrative purposes, one study in our meta‐analytic sample (Senn & Forrest, 2016) reported a preintervention mean score of 62.47 (*SD* = 20.58) on the Bystander Efficacy Scale, with a total possible score of 100 and lower scores representing greater bystander efficacy. Considering that the average effect size for bystander efficacy at immediate posttest in our sample was 0.45, extrapolation suggests that participating in a bystander program decreased the mean bystander efficacy score from 62.47 to 53.21 (i.e., (62.47 − [0.45 × 20.58]) = 53.21), a desirable change of approximately 9 points on a 100‐point scale.

Program effects on identifying a situation as appropriate for intervention were significant but were smaller at later follow‐up waves. At immediate posttest measures of this outcome were almost six‐tenths of a standard deviation higher for bystander program participants than for the comparison group. By 1‐ to 4‐month follow‐up this difference was four‐tenths. Only one study measured identifying a situation as appropriate for intervention past 6 months follow‐up. To put the immediate posttest findings into context, one study in our meta‐analytic sample (Addison, 2015) reported a preintervention mean rating of 5.94 (*SD* = 1.11) regarding responses to a vignette depicting a scenario in which a young woman was too inebriated to consent to sexual activity. The total possible score was 7, with higher scores indicating stronger agreement that the scenario depicted in the vignette warranted intervention. Considering that the average effect size for this outcome was 0.57 at immediate posttest, extrapolation suggests that participating in a bystander program increased the mean rating from 5.94 to 6.57 (i.e., (5.94 + [0.57 * 1.11]) = 6.57), a change of approximately two‐thirds a point on a 7‐point scale.

For three of the attitude/knowledge outcomes, effects were nonsignificant at immediate posttest, but significant at the 1‐ to 4‐month post‐intervention follow‐up period. This was the case for taking responsibility to intervene, knowing strategies for intervening, and intentions to intervene. At 1‐ to 4‐month follow‐up, relative to the comparison group, bystander program participants reported greater responsibility to intervene (difference of approximately one‐third a standard deviation). To put these findings into context, one study in our meta‐analytic sample (Senn & Forrest, 2016) reported a preintervention mean score of 3.51 (*SD* = 1.13) on a measure of responsibility to intervene, with a total possible score of 7 and lower scores indicating greater responsibility. Considering that the average effect size for this outcome was 0.32, extrapolation suggests that participating in a bystander program decreased the mean score on this outcome from 3.51 to 3.15 (i.e., (3.51 + [0.32 * 1.13]) = 3.15), a desirable change of approximately four‐tenths a point on a 7‐point scale.

At one‐to‐four‐month follow‐up, relative to the comparison group, bystander program participants reported higher measures of knowing strategies for intervening (approximately six‐tenths of a standard deviation higher). However, this effect was only based on one study.

At one‐ to‐four‐month follow‐up, relative to the comparison group, bystander program participants reported higher measures of intentions to intervene (approximately four‐tenths a standard deviation higher). To put these findings into context, one study in our meta‐analytic sample (Salazar et al., 2014) reported a preintervention mean score of 52.09 (*SD* = 12.78) on the Bystander Intention Scale, with a total possible score of 75 and higher scores indicating greater intentions. Considering that the average effect size for this outcome was 0.41 at the one‐to‐four‐months follow‐up wave, extrapolation suggests that participating in a bystander program increased the mean score on this outcome from 52.09 to 57.33 (i.e., 52.09 + [0.41 × 12.78]) = 57.33), a desirable change of approximately 5 points on a 75‐point scale. Significant effects for this outcome were also observed at the 6 months to 1‐year follow‐up wave (approximately one‐fourth a standard deviation). Considering the effect size at this follow‐up wave was 0.23, participation in a bystander program increased the mean bystander intention score from 52.09 to 55.03 between preintervention and the 6‐month to 1 year follow‐up (i.e., 52.09 + [0.23 × 12.78]) = 55.03), a change of approximately 3 points on a 75‐point scale. Additionally, the mean score on bystander intentions decreased from 57.33 to 55.03 between one‐to‐four‐month follow‐up and 6 months to 1‐year follow‐up.

The fact that significant effects were delayed until 1‐ to 4‐month follow‐up for three attitude/knowledge outcomes may indicate that participants' knowledge and attitudes shifted over time or that the programs required a bit of rumination and reflection to take effect. We found limited or no evidence of intervention effects on gender attitudes, victim empathy, date rape attitudes, and noticing sexual assault.

##### 10.1.1.2 Behavior

Our results indicated that bystander programs have a desirable effect on bystander intervention. At 1‐ to 4‐month follow‐up measures of bystander intervention were approximately one‐fourth a standard deviation higher for bystander program participants than for the comparison group. To contextualize, one study in our meta‐analytic sample (Jouriles et al., 2016a) reported a pre‐intervention mean score of 27.95 (*SD* = 19.02) on the Bystander Intervention Scale, with a total possible score of 49 and higher scores indicating greater intervention behavior over the past month. Considering that the average effect size for bystander intervention at 1‐ to 4‐month follow‐up was 0.27, then extrapolation suggests that participation in a bystander intervention program increased bystander intervention scores from 27.95 to 33.09 (i.e., (27.95 + [0.27 * 19.02]) = 33.09), indicating that participants engaged in approximately five additional acts of intervention in the past month (relative to pretest behavior). However, this effect was nonsignificant at 6 months post‐intervention.

It is noteworthy that bystander programs did not have a significant effect on sexual assault perpetration. This finding seems to belie the original intent of some of the earliest bystander programs, which aimed to reduce sexual violence perpetration by approaching young people (young men especially) as allies in preventing violence against women, rather than as potential perpetrators of violence. This nonthreatening approach was anticipated to be more effective at reducing violence against women than traditional victim/perpetrator‐focused models, which may risk young men's defensiveness or backlash (Katz, 1995; Messner, 2015). Instead, evidence from our review indicates that, although bystander programs may have a meaningful, short‐term effect on bystander intervention behavior, there is no evidence that the programs have a similar effect on sexual assault perpetration. Thus, it appears that bystander program participants interpret program information from the perspective of potential allies rather than potential perpetrators.

#### 10.1.2 Objective 2: Effects for different participant profiles

We had planned to conduct moderator analyses to assess any differential effects of bystander programs on outcomes based on the following participant characteristics: mean age of the sample, education level of the sample, proportion of males/females in the sample, proportion of fraternity/sorority members in the sample, and proportion of athletic team members in the sample. Our review only produced a sufficient number of studies (*n* ≥ 10) to conduct such moderator analyses for the bystander intervention behavior outcome. Those results provided no evidence that mean age, education level, or proportion of males/female were associated with the magnitude of program effects on bystander intervention behavior. We were unable to conduct moderator analyses for proportion of fraternity/sorority members in the sample and proportion of athletes in the sample, as these variables were rarely or never reported.

#### 10.1.3 Objective 3: Effects based on gendered content/implementation

We conducted moderator analyses to assess any differential effects of bystander programs on measured outcomes based on (a) the gender of perpetrators and victims in specific bystander programs and (b) whether programs were implemented in mixed‐ or single‐sex settings. Our review only produced a sufficient number of studies (*n* ≥ 10) to conduct such moderator analyses for the bystander intervention behavior outcome. The results provided no evidence that these measures were associated with the magnitude of program effects on bystander intervention behavior.

### 10.2 Overall completeness and applicability of evidence

The thorough nature of our systematic literature search process (e.g., searching electronic databases, ClinicalTrials.gov, conference proceedings, organization websites, reference lists of review articles and eligible reports, tables of contents of relevant journals, CVs and websites of primary authors of eligible studies, and forward citation searching of eligible studies) helped ensure that all relevant studies were identified for consideration in this review. Nonetheless, there were three rogue reports that we were unable to locate. Readers should be mindful of this when interpreting findings from this review.

Readers should also be mindful that, because bystander sexual assault prevention programs are not standardized, the summarized research employed a variety of modalities, program curricula, and delivery methods. In order to be included in this systematic review and meta‐analysis, all programs had to include a bystander intervention component, but there were no other restrictions or limitations on programs. Readers should therefore consider that our syntheses include different types of programs. Although this does not necessarily limit the applicability or generalizability of the systematic review and meta‐analysis, readers should be cognizant that a variety of bystander intervention programs may be included in each quantitative synthesis.

Although our systematic literature search identified 27 eligible studies, only 13 reported bystander behavior and only six reported sexual assault. Thus, results for these behavioral outcomes may be considered tentative, as an updated review that includes more studies may produce different findings. Additionally, included studies rarely reported some of the moderators that we identified in our protocol (i.e., fraternity/sorority membership and athletic team membership) and the vast majority (i.e., 25 of 27) were conducted in the United States. Thus, we do not know how well fraternity/sorority members and athletic team members were represented in the sample and we do know that participants from the United States were over‐represented.

This paucity of international research highlights a clear need for further study of bystander programs in a global context and serves to underscore the necessity of forming a broader evidence base. Readers should be cautioned that the findings of this review may not generalize to all contexts, especially those that are demographically different from the United States.

Finally, no eligible studies examined sexual assault in lesbian, gay, bisexual, or queer relationships or interactions, and programs reported in the literature did not make clear that they mentioned or addressed potential sexual assault in nonheterosexual relationships or interactions. Further, eligible studies did not indicate that they provided any particular or tailored strategies for being a bystander in nonheterosexual situations.

### 10.3 Quality of the evidence

Although we deemed a large number of studies to be ineligible based on methodological quality, the large body of extant research on bystander program permitted the identification of 27 independent studies that met all inclusion criteria for this review. Of these 27 studies, 21 were RCTs (i.e., 12 randomized at the individual level and 9 randomized at the group level) and 6 were high‐quality quasi‐experimental studies.

Risk of bias for the RCTs in this review was relatively low; however, there were some issues that must be noted. First, we coded approximately 95% of the sample of RCTs as exhibiting high risk of bias pertaining to blinding of outcome assessments. This was due to the frequent reporting of self‐report outcomes. Second, we coded approximately 80% of the sample of RCTs as exhibiting high risk of bias in some “other” domain. Such designation was typically the result of study authors evaluating programs that they designed themselves. Risk of bias for the nine non‐RCT studies was typically low or unclear. The latter designation was typically a result of study authors' failure to report our prespecified confounding variables broken down by treatment and comparison groups.

### 10.4 Limitations and potential biases in the review process

As a whole, the existing evidence base for this review (*N* = 27) was fairly strong methodologically, but the relatively small number of eligible studies in each meta‐analysis precluded our analysis of important sources of heterogeneity. That is, only one meta‐analysis contained sufficient studies (*n* ≥ 10) to conduct moderator analyses and explore issues stemming from risk of bias or publication/small study bias. Future accumulation of high‐quality research should permit such analyses in updated reviews. Additionally, the dearth of non‐US research is a limitation to the generalizability of the findings.

### 10.5 Agreements and disagreements with other studies or reviews

To date, there is only one existing meta‐analysis examining the effects of bystander programs. In what they called an “initial” meta‐analysis of experimental and quasi‐experimental studies (published through 2011), Katz & Moore (2013) found moderate effects of bystander programs on participants' self‐efficacy and intentions to intervene, and small (but significant) effects on bystander behavior, rape‐supportive attitudes, and rape proclivity (but not perpetration). Effects were generally stronger among younger samples and samples containing a higher percentage of males.

The main findings from the current review are consistent with those reported by Katz and Moore. Specifically, like Katz and Moore's analysis, our review findings indicated that bystander programs have beneficial effects on bystander efficacy and intentions. Additionally, we found beneficial effects on bystander behavior (i.e., bystander intervention) and rape supportive attitudes (i.e., rape myth acceptance). Unlike Katz and Moore, however, we did not analyze rape proclivity outcomes; but similar to Katz and Moore, we found no evidence of an effect on sexual assault perpetration.

Katz and Moore also conducted moderator analyses to assess differential effects of bystander programs on (a) bystander efficacy and (b) bystander intentions. They reported that program effects on bystander efficacy were stronger among younger participants and that effects on bystander intentions were stronger among samples containing a higher proportion of males. Our moderator analyses provided no evidence that participant age and proportion of males were predictors of program effects. However, we were unable to explore these moderators in depth given the small number of studies included in each meta‐analysis.

Although findings from our meta‐analysis are largely similar to those of Katz and Moore, our analysis advances our understanding of the evidence base for bystander programs in two important ways. First, whereas Katz and Moore's analysis focused on bystander programs implemented with college students, our analysis focused on programs implemented with both college students and adolescents. Given that our moderator analyses indicated age and education level were not significant moderators of program effects on bystander intervention, our findings for this outcome may be representative of both college students and adolescents. However, findings from these moderator analyses should be interpreted as preliminary, given the small number of studies reporting bystander intervention.

Second, whereas Katz and Moore did not evaluate program content/implementation as a moderator of program effects, we were able to examine the influence of such variables on an important outcome. We found that (a) sex of perpetrators/victims in bystander programs and (b) whether programs were implemented in mixed‐ or single‐group settings were not significant moderators of program effects on bystander intervention. Again, however, findings from these moderator analyses should be interpreted as preliminary given the small number of studies reporting bystander intervention.

## 11 AUTHORS' CONCLUSIONS

### 11.1 Implications for practice and policy

The United States 2013 Campus SaVE Act requires postsecondary educational institutions participating in Title IX financial aid programs to provide incoming college students with primary prevention and awareness programs addressing sexual violence. The Campus SaVE Act mandates that these programs include a component on bystander intervention. Currently, there is no comparable legislation regarding sexual assault among adolescents (e.g., mandating bystander programs in secondary schools). This is an unfortunate oversight, as adolescents who experience sexual assault are at an increased risk of repeated victimization in young adulthood (Cui et al., 2013). Thus, the implementation of bystander programs in secondary schools not only has the potential to reduce sexual assault among adolescents, but may also have the long‐term potential to reduce sexual assault on college campuses.

Findings from this review indicate that bystander programs have significant beneficial effects on bystander intervention behavior. This provides important evidence of the effectiveness of mandated programs on college campuses. Additionally, the fact that our (preliminary) moderator analyses found program effects on bystander intervention to be similar for adolescents and college students suggests early implementation of bystander programs (i.e., in secondary schools with adolescents) may be warranted.

Importantly, although we found that bystander programs had a significant beneficial effect on bystander intervention behavior, we found no evidence that these programs had an effect on participants' sexual assault perpetration. Bystander programs may therefore be appropriate for targeting bystander behavior, but may not be appropriate for targeting the behavior of potential perpetrators. Additionally, effects of bystander programs on bystander intervention behavior diminished by 6‐month post‐intervention. Thus, programs effects may be prolonged by the implementation of booster sessions conducted prior to 6 months post‐intervention.

### 11.2 Implications for research

Findings from this review suggest there is a fairly strong body of research assessing the effects of bystander programs on attitudes and behaviors. However, there are a couple of important questions worth further exploration.

First, according to one prominent logical model, bystander programs promote bystander intervention by fostering prerequisite knowledge and attitudes (Burn, 2009). Our meta‐analysis provides inconsistent evidence of the effects of bystander programs on knowledge and attitudes, but promising evidence of short‐term effects on bystander intervention. This casts uncertainty around the proposed relationship between knowledge/attitudes and bystander behavior. Although we were unable to assess these issues in the current review, this will be an important direction for future research. Our understanding of the causal mechanisms of program effects on bystander behavior would benefit from further analysis (e.g., path analysis mapping relationships between specific knowledge/attitude effects and bystander intervention).

Second, bystander programs exhibit a great deal of content variability, most notably in framing sexual assault as a gendered or gender‐neutral problem. That is, bystander programs tend to adopt one of two main approaches to addressing sexual assault: (a) presenting sexual assault as a gendered problem (overwhelmingly affecting women) or (b) presenting sexual assault as a gender‐neutral problem (affecting women and men alike). Differential effects of these two types of programs remain largely unexamined. Our analysis indicated that (a) the sex of victims/perpetrators (i.e., portrayed in programs as gender neutral or male perpetrator and female victim) and (b) whether programs were implemented in mixed‐ or single‐sex settings were not significant moderators of program effects on bystander intervention. However, these findings are limited to a single outcome and they should be considered preliminary, as they are based on a small sample (*n* = 11). Our understanding of the differential effects of gendered versus gender neutral programs would benefit from the design and implementation of high‐quality primary studies that make direct comparisons between these two types of programs (e.g., RCTs comparing the effects of two active treatment arms that differ in their gendered approach).

Finally, our systematic review and meta‐analysis demonstrate the lack of global evidence concerning bystander program effectiveness. Our understanding of bystander programs' generalizability to non‐US contexts would be greatly enhanced by high quality research conducted across the world.

## FUNDING INFORMATION

This review was supported by a grant from Campbell (CSR1.60).

## CONFLICT OF INTERESTS

The authors declare that there is no conflict of interests.

## AUTHOR CONTRIBUTIONS

Members of the research team responsible for core areas of the review are as follows: Content: H. H. K.; Systematic review methods: H. H. K., R. A. M., and E. E. T.‐S.; Statistical analysis: H. H. K.; Information retrieval: H. H. K. and R. A. M. Dr Kettrey, the lead review author, coordinated the review team and assumed responsibility for the implementation of the project throughout its duration. Specific tasks included compiling the sample of research reports, creating the database, coding studies, analyzing data, and preparing the Campbell Review. Dr Marx, the second review author, collaborated closely with Dr Kettrey to compile the sample of research reports, code studies, and make methodological decisions throughout the duration of the project. Dr Tanner‐Smith, the third review author, provided methodological guidance and mentorship to Dr Kettrey and Dr Marx throughout all phases of data collection and analysis.

## Supporting information

Supporting information
