# Supplementary material for: Effects of bystander programs on the prevention of sexual assault among adolescents and college students: A systematic review
Source: Campbell Syst Rev. 2019 Jul 18;15(1-2):e1013. doi: 10.4073/csr.2019.1 (PMC8356505; doi:10.4073/csr.2019.1)
Supplement: Supplementary file 1 — Supporting information [file CL2-15-e1013-s001.docx]

# Appendix: Codebook

Bystander INTERVENTION

META-ANALYSIS

CODING MANUAL

Last Updated May 17, 2017

Table of Contents

ELIGIBILITY CRITERIA 133

FULL-TEXT CODING 136

Study Level 136

Intervention and Comparison Groups 146

Outcomes 152

Effect Sizes 154

# Eligibility criteria

Intervention

1. Eligible intervention programs are those that approach participants as allies in preventing and/or alleviating intimate partner violence (IPV) and/or sexual assault among adolescents and/or college students. (NOTE: For the purposes of this meta-analysis, IPV includes physical or sexual violence perpetrated by an intimate partner. Sexual assault refers to sexual violence that may be perpetrated by an intimate partner, acquaintance, or stranger). Some part of the program must focus on ways that cultivate a willingness for a person to respond to others who are at risk for IPV or sexual assault. All delivery formats are eligible for inclusion (e.g., in-person training sessions, video programs, web-based training, advertising/poster campaigns). There are no treatment duration criteria for inclusion. The following interventions are ineligible:
   1. Studies that report bystander outcomes but do not meet the aforementioned intervention criteria are not eligible for inclusion. For example, studies that assess bystander outcomes of general IPV/sexual assault prevention programs that contain NO bystander content.
   2. Studies that assess outcomes of programs that aim to facilitate pro-social bystander behavior, but that do not explicitly include a component addressing IPV and/or sexual assault (e.g., programs that use a bystander approach to prevent bullying) are not eligible.

COMPARISON

1. Eligible comparison groups must receive no intervention services targeting bystander attitudes/behavior, IPV, or sexual assault. Thus, treatment-treatment studies that compare individuals assigned to a bystander program with individuals assigned to a general IPV or sexual assault prevention program are not eligible for inclusion. However, eligible comparison groups may receive a sham or attention treatment that is expected to have no effect on bystander outcomes or attitudes/behaviors regarding IPV or sexual assault (e.g., nutrition or health education).

PARTICIPANTS AND SETTING

1. The intervention must be implemented with adolescents and/or college students in educational settings. Eligible participants include adolescents enrolled in grades 7 through 12 and college students enrolled in any type of undergraduate postsecondary educational institution. Studies that assess bystander programs implemented with adolescents and young adults outside of educational institutions (e.g., in community settings, military settings) are ineligible for inclusion in the review.
   1. Eligible studies include those that report on general samples of adolescents and/or college students as well as studies using specialized samples such as those primarily consisting of college athletes, fraternity/sorority members, and single-sex samples.
   2. Study samples primarily consisting of post-graduate students will be ineligible for inclusion; the mean age of samples may be no greater than 25 to be included in the review.

RESEARCH DESIGN

1. Eligible studies must use an experimental or controlled quasi-experimental research design to compare an intervention group (e.g., students assigned to complete a bystander program) with a comparison group (e.g., students not assigned to complete a bystander program). The following are eligible designs:
   1. Randomized controlled trials: Studies in which individuals, classrooms, schools, or other groups are randomly assigned to intervention and comparison conditions.
   2. Quasi-randomized controlled trials: Studies where assignment to conditions is quasi-random, for example, by birth date, day of week, student identification number, month, or some other alternation method.
   3. Controlled quasi-experimental designs: Studies where participants are not assigned to conditions randomly or quasi-randomly (e.g., participants self-select into groups). Given the potential selection biases inherent in these controlled quasi-experimental designs, we will only include those that also meet one of the following criteria:
      1. Regression discontinuity designs: Studies that use a cutoff on a forcing variable to assign participants to intervention and comparison groups, and assess program impacts around the cutoff of the forcing variable.
      2. Studies that use propensity score or other matching procedures to create a matched sample of participants in the intervention and comparison groups. To be eligible for inclusion, these studies must also provide enough statistical information to permit estimation of pretest effect sizes for the matched groups (NOTE: Pretest effect sizes refer to pre-intervention measures of an outcome variable. Pre-intervention measures that are not reported as outcomes are group equivalence measures).
      3. For studies where participants in the intervention and comparison groups are not matched, enough statistical information must be reported that will permit us to estimate pretest effect sizes for at least one outcome measure. Pretest must be administered to the treatment and comparison group at the same point in time.

SAMPLE SIZES

1. Intervention and control groups must each contain at least 10 individuals at the time of assignment to study conditions.

OUTCOME MEASURES

1. Eligible studies must report the effects of bystander programs on at least one of the following primary outcomes:
   1. General attitudes toward IPV and/or sexual assault and victims (e.g., victim empathy, rape myth acceptance).
   2. Self-efficacy with regard to bystander intervention (e.g., measures of confidence in respondents’ ability to intervene).
   3. Intentions to intervene when witnessing instances or warning signs of IPV and/or sexual assault.
   4. Actual intervention behavior when witnessing instances or warning signs of IPV and/or sexual assault.
   5. Perpetration of IPV and/or sexual assault.

**NOTES:** (1) Studies that report outcomes by breakout groups (e.g., report findings separately for males/females, fraternity/nonfraternity, etc.) are not eligible unless findings are reported such that outcomes for breakout groups can be combined. (2) Studies must assess at least one outcome pertinent to sexual assault to be included in the main Campbell review and meta-analysis. Studies that only assess physical violence outcomes (e.g., evaluations of IPV bystander programs that measure physical violence outcomes, but not sexual violence outcomes) will be coded and set aside for separate analysis. These must be marked as **“Physical Violence Only”** on the eligibility screen in FileMaker. There are no other restrictions on eligibility. Studies that meet all eligibility criteria but do not provide sufficient information for calculating effect sizes are still eligible for inclusion and will be coded (i.e., the study will be coded on all items except the effect sizes).

# Full-text coding

## Study level

**[studyid]**

Study identification number. The “unit” you will code here consists of a study, i.e., one research investigation of a defined subject sample or subsamples compared to each other, and the treatments, measures, and statistical analyses applied to them. Sometimes there are several different reports about a single study. In such cases, the coding should be done from the full set of relevant reports, using whichever report is best for each item to be coded; BE SURE YOU HAVE THE FULL SET OF RELEVANT REPORTS BEFORE BEGINNING TO CODE. Sometimes a single report describes more than one study sample, e.g., evaluations at three separate sites. In these cases, each study sample will have a unique study identification number and each study should be coded separately as if it had been described in a separate report.

Each study has its own study identification number, or StudyID (e.g., 619). Each report also has an identification number (e.g., 619.01), which you will find in the FileMaker bibliography. The ReportID has two parts; the part before the decimal is the StudyID, and the part after the decimal is used to distinguish the reports within a study. (These two types of ID numbers, along with bibliographic information, are assigned and tracked using the bibliography.) When coding, use the study ID (e.g., 619) to refer to the study as a whole, and use the appropriate report ID (e.g., 619.01) when referring to an individual report.

**[coder]**

Coder's initials

***HEADER VARIABLES***

**[pub.year]**

Year of publication for the primary report (four digits). If more than one report exists, choose the date for the report that provides the effect sizes. If effect sizes come from more than one report, choose the earliest date.

**[publication]**

Type of publication for the primary report. If you are using more than one type of publication to code your study, choose the publication that supplies the effect sizes (in cases where more than one report provides effect sizes, choose a “peer reviewed” choice over another option, or choose the report that provides the most effect sizes).

1. Journal Article
2. Book or book chapter
3. Dissertation
4. MA/MS Thesis
5. Private report
6. Government report
7. Conference paper
8. Other
9. Unclear

**[peer]**

Is this a peer-reviewed publication (primary report only)?

1. Yes
2. No
3. Unclear

**[funded]**

Do the authors declare any funding?

1. Yes
2. No
3. Unclear

**[researcher_role]**

Did one or more of the researchers assessing outcomes of the intervention program (i.e., authors of the research report) play a significant role in developing the program?

1. Yes – One or more researchers developed the intervention program.
2. No – Researchers are independent evaluators.
3. Unclear/Cannot tell.

The following are the developers of some potentially eligible bystander (or peer/social network-based) programs. Some of the programs below were developed by individuals (e.g., academics) and others were developed by organizations. In the latter case, any research report authored by a representative of the developer/organization should be coded as “1.” Less popular programs that do not appear on this list may require a careful reading of the text (e.g., the authors will likely cite the program in the evaluation report) and/or some additional research.

- **Bringing in the Bystander:** Developed by Victoria Banyard, Mary Moynihan, Elizabeth Plante (University of New Hampshire).
- **Circle of 6 (potentially ineligible):** Developed by Nancy Schwartzman, Thomas Cabus. See list of advisory board members (http://www.circleof6app.com/about/#member2).
- **Coaching Boys into Men:** Developed by Futures without Violence. See list of board and staff members (https://www.futureswithoutviolence.org/about-us/board-and-staff/).
- **Green Dot:** Developed by Dorthy Edwards.
- **Hollaback (potentially ineligible):** Developed by Emily May.
- **InterACT:** Marc Rich may or may not be the developer, but he has a conflict of interest, as he directs this program/troupe.
- **Know Your Power:** This is the campaign companion to Bringing in the Bystander. Developed by Sharyn Potter, Jane Stapleton, Mary Moynihan (University of New Hampshire).
- **Men Can Stop Rape:** Developed by John Stoltenberg. See MCSR staff members (http://www.mencanstoprape.org/Our-Staff/) and board members (http://www.mencanstoprape.org/Our-Board/).
- **Mentors in Violence Prevention (MVP):** Developed by Jackson Katz.
- **Red Flag Campaign:** Developed by Virginia Sexual and Domestic Violence Action Alliance. See list of governing body members (http://www.vsdvalliance.org/#/aboutcontact-us/governing-body).
- **Speak Up Speak Out (potentially ineligible):** Developed by Maryland Coalition Against Sexual Assault.
- **Step Up!:** Developed by Becky Bell.
- **That’s Not Cool:** Developed by Futures without Violence. See list of board and staff members (https://www.futureswithoutviolence.org/about-us/board-and-staff/).
- **The Men’s Program (How to Help a Sexual Assault Survivor):** Developed by John Foubert (Oklahoma State University).
- **The Women’s Program**: Developed by John Foubert (Oklahoma State University).
- **Where Do You Stand:** This is an ad campaign developed by Men Can Stop Rape. MCSR was developed by John Stoltenberg. See MCSR staff members (http://www.mencanstoprape.org/Our-Staff/) and board members (http://www.mencanstoprape.org/Our-Board/).
- **White Ribbon Campaign:** Developed by Michael Kaufman, Jack Layton.

***Group Identification and Selection***

At this stage, you will need to identify the groups in the study for which effect size statistics can be computed. Note that for any group comparison coding, the two groups involved must be from the same experiment or quasi-experiment; that is, they must have been involved in the same randomization, matching, etc. from the same design. If two or more experiments or quasi-experiments are presented in the same report, each must be handled separately.

**[txa-d]** Write in Name

Treatment Groups 1-4 __________________________________

**[cta-d]** Write in Name

Comparison Groups 1-4 __________________________________

***STUDY CONTEXT***

**[country]**

Country in which the study was conducted:

1. United States
2. Canada
3. Mexico
4. Australia
5. European Nation
6. Other

**[state]**

State in which the study was conducted.

1. Alabama
2. ….
3. …
4. District of Columbia
5. Single state (unspecified)
6. Multiple states (unspecified)
7. NA – Conducted outside US

**[study.year]**

Year(s) when study was conducted. Code NA if cannot tell.

**[ed.setting]**

Type of educational setting in which the study was conducted:

1. Secondary school (grades 7-12)
2. College or university
3. Unclear

***Study Design and Methods***

**[design]**

Method of assignment to groups. This item focuses on the initial method of assignment to groups regardless of subsequent degradations due to attrition, refusal, etc. prior to treatment onset. These latter situations are coded elsewhere.

Random or near-random:

1. Random assignment at the individual level: Individual participants are randomly assigned to conditions. In some cases random assignment may be done after individuals have been matched or blocked.
2. Random assignment by group: Intact groups such as classrooms are assigned.
3. Regression discontinuity design: Individuals are assigned to groups based on a cut-off score of a forcing variable (i.e., pretest or risk factor measure). Note that this design will be rare.
4. Quasi-randomized procedure presumed to produce comparable groups: This applies to groups which have individuals assigned by some naturally occurring process that is apparently random, e.g. alternation, date of birth, medical record number. The key here is that the procedure used to select groups is not strictly random, but the method of allocation should not create nonequivalence between groups.

Non-random, but matched or statistically controlled:

Note: Matching refers to the process by which individuals are selected for conditions (e.g., treatment and comparison) in a manner that ensures that the individuals in one group are matched on certain relevant characteristics in the other group. Comparing the characteristics of the groups AFTER they have been assigned to experimental conditions does NOT constitute matching.

1. Matched individually, through sampling, on one or more pretest measures (i.e., attitudes toward IPV/sexual assault or victims, bystander self-efficacy, bystander intentions, bystander behavior, IPV/sexual assault perpetration).
2. Participants in the intervention and comparison groups are not matched, but enough statistical information is provided to permit the estimation of pretest effect sizes for at least one outcome measure (i.e., attitudes toward IPV/sexual assault or victims, bystander self-efficacy, bystander intentions, bystander behavior, IPV/sexual assault perpetration).
3. Matched at a larger group level; that is, intact groups were matched on their means for one or more pretest measures (i.e., attitudes toward IPV/sexual assault or victims, bystander self-efficacy, bystander intentions, bystander behavior, IPV/sexual assault perpetration). In these cases the mean pretest measures of the groups are similar, but each subject in one group has not been individually matched to a subject in the other group on age.

**[matched.var.list]**

Please list all of the variables used in the matching and/or statistical controls.

**[m]**

For cluster randomized trials, please enter the average cluster size (i.e., average number of participants in each cluster). Code -9 for cannot tell. Code -8 for Not applicable.

**[attrf_o]**

What is the overall attrition rate (across all groups) in the study between the time of assignment to groups to the first follow-up? This item refers to overall attrition in the study; more detailed attrition calculations will be estimated using the assigned and observed sample sizes coded in the effect size section.

**[attrl_o]**

What is the overall attrition rate (across all groups) in the study between the time of assignment to groups to the last follow-up? Again, this item refers to overall attrition in the study; more detailed attrition calculations will be estimated using the assigned and observed sample sizes coded in the effect size section.

**[itt]**

Did the authors use an intent-to-treat (ITT) analysis? Intent-to-treat analysis refers to situations where researchers ‘analyze as randomized’, meaning that all individuals who were randomized to the intervention/control groups are included in the final outcome analysis, regardless of whether they actually attended/completed the intervention. Note, that it is possible for a study to conduct an ITT analysis even if they have attrition, as long as they had intended to include any non-completers in their final model.

1. Yes – explicit ITT analysis
2. No – completer analysis, TOT analysis
3. Cannot tell

**[missdata]**

How did the authors handle missing data in their analysis?

1. Listwise deletion
2. Pairwise deletion
3. Mean or mode imputation
4. Single regression imputation
5. Dummy variable approach (imputed value at zero with dummy variable)
6. Multiple imputation
7. Full information maximum likelihood (FIML)
8. Other method
9. Not applicable – no missing data
10. Cannot tell

***RISK OF BIAS: NON-EXPERIMENTAL STUDIES***

Although these risk of bias variables will largely be used in analyses of non-experimental studies, please code them for all eligible studies, regardless of research design.

**[rob_ne_confounding]**

Describe any risk of bias for non-experimental studies based on pre-specified confounding factors (i.e., gender, fraternity/sorority membership, athletic team membership, pre-intervention attitudes, pre-intervention bystander measures, and prior sexual assault victimization). That is, please note if a considerable percentage of participants exhibited any of these confounding variables (e.g., if the study was implemented with a sample of fraternity/sorority members).

**[rob_ne_cointerventions]**

Describe any risk of bias for non-experimental studies based on pre-specified co-interventions (i.e, general sexual assault prevention programs, IPV prevention programs, and general bystander programs). That is, please note if there is reason to believe participants had previous or concurrent exposure to any potentially confounding co-interventions.

***RISK OF BIAS: EXPERIMENTAL STUDIES***

Although these risk of bias variables will largely be used in analyses of experimental studies, please code them for all eligible studies, regardless of research design.

**[rob_sg]**

**Random Sequence Generation:** What is the risk of ***sequence generation*** due to inadequate generation of a randomized sequence?

1. Low risk. The investigators describe a random component in the sequences generation process such as;
   1. Referring to a random number table;
   2. Using a computer random number generator;
   3. Coin tossing;
   4. Shuffling cards or envelopes;
   5. Throwing dice;
   6. Drawing of lots;
   7. Minimization (may be implemented without a random element, and this is considered equivalent to being random).
2. High risk. The investigators describe a non-random component in the sequence generation process. Usually, the description would involve some systematic non-random approach, for example:
   1. Sequence generated by odd/even birth dates;
   2. Sequence generated by some rule based on date (or day) of admission;
   3. Sequence generated by some rule based on some sort of (e.g., hospital/clinic) record number.

Other non-random approaches happen much less frequently than the systematic approaches mentioned above and tend to be obvious. They usually involve judgment or some method of non-random categorization of participants, for example:

1. Allocation by judgment of the clinician (or teacher or practitioner);
2. Allocation by preference of the participant;
3. Allocation based on results of a laboratory test or series of tests;
4. Allocation by availability of the intervention.

By definition, any quasi-experimental design where participants self-select into conditions is at high risk of bias.

1. Unclear risk of bias. Insufficient information is provided about the sequence generation process to permit judgement of low or high risk.

**[rob_sg_text]**

Provide a description of the information used to code the risk of bias due to sequence generation.

**[rob_allocation]**

**Allocation Concealment:** What is the risk of bias due to ***allocation concealment*** (inadequate concealment of allocations prior to assignment)? Note that most studies will not report information on allocation concealment.

1. Low risk. Participants and investigators enrolling participants could not foresee assignment because one of the following, or an equivalent method, was used to conceal allocation:
   1. Central allocation (including telephone, web-based, and pharmacy-controlled randomization);
   2. Sequentially numbered drug containers of identical appearance;
   3. Sequentially numbered, opaque, sealed envelopes
2. High risk. Participants or investigators enrolling participants could possibly foresee assignments and thus introduce bias, such as allocation based on:
   1. Using an open random allocation schedule (e.g., a list of random numbers);
   2. Assignment envelopes were used without appropriate safeguards (i.e., if envelopes were unsealed or non-opaque or not sequentially numbered);
   3. Alternation or rotation;
   4. Date of birth;
   5. Case record number;
   6. Any other explicitly unconcealed procedure.
3. Unclear risk. Insufficient information to permit judgment of ‘low risk’ or ‘high risk.’ This is usually the case if the method of concealment is not described or not described in sufficient detail to allow a definite judgement – for example if the use of assignment envelopes is described, but it remains unclear whether envelopes were sequentially numbered, opaque or sealed.

**[rob_allocation_text]**

Provide a description of the information used to code the risk of bias due to allocation concealment.

**[rob_blinding_part]**

**Blinding of Participants and Personnel:** What is the risk of bias due to ***knowledge*** of the allocated interventions ***by participants and personnel***?

1. Low risk. Any one of the following:
   1. No blinding or incomplete blinding, but the review authors judge that the outcome measurement is not likely to be influenced by lack of blinding;
   2. Blinding of participants and key study personnel ensured, and unlikely that the blinding could have been broken.
2. High risk. Any one of the following:
   1. No blinding or incomplete blinding, and the outcome measurement is likely to be influenced by lack of blinding;
   2. Blinding of key study participants and personnel attempted, but likely that the blinding could have been broken, and the outcome measurement is likely to be influenced by lack of blinding.
3. Unclear risk. Insufficient information to permit judgment of ‘low risk’ or ‘high risk.’

**[rob_blinding_part_text]**

Provide a description of the information used to code the risk of bias due to insufficient blinding of participants and personnel.

**[rob_blinding_assess]**

**Blinding of Outcome Assessment:** What is the risk of bias due to ***knowledge*** of the allocated interventions ***by outcome assessors***?

1. Low risk. Any one of the following:
   1. No blinding of outcome assessment, but the review authors judge that the outcome measurement is not likely to be influenced by lack of blinding;
   2. Blinding of outcome assessment ensured, and unlikely that the blinding could have been broken
2. High risk. Any one of the following:
   1. No blinding of outcome assessment, and the outcome measurement is likely to be influenced by lack of blinding;
   2. Blinding of outcome assessment, but likely that the blinding could have been broken, and the outcome measurement is likely to be influenced by lack of blinding.
   3. All outcomes are self-reported outcomes (which inherently are NOT blinded)
3. Unclear risk. Insufficient information to permit judgment of ‘low risk’ or ‘high risk.’

**[rob_blinding_assess_text]**

Provide a description of the information used to code the risk of bias due to insufficient blinding of outcome assessment.

**[rob_incomplete]**

**Incomplete Outcome Data:** What is the risk of attrition bias due to amount, nature, or handling of ***incomplete outcome data***?

1. Low risk. Any one of the following:
   1. No missing outcome data;
   2. Reasons for missing outcome data unlikely to be related to true outcome (for survival data, censoring unlikely to be introducing bias);
   3. Missing outcome data balanced in numbers across intervention groups, with similar reasons for missing data across groups;
   4. For dichotomous outcome data, the proportion of missing outcomes compared with observed event risk not enough to have a clinically relevant impact on the intervention effect estimate;
   5. For continuous outcome data, plausible effect size (difference in means or standardized difference in means) among missing outcomes not enough to have a clinically relevant impact on observed effect size;
   6. Missing data have been imputed using appropriate methods.
2. High risk. Any one of the following:
   1. Reason for missing outcome data likely to be related to true outcome, with either imbalance in numbers or reasons for missing data across intervention groups;
   2. For dichotomous outcome data, the proportion of missing outcomes compared with observed event risk enough to induce clinically relevant bias in intervention effect estimate;
   3. For continuous outcome data, plausible effect size (difference in means or standardized difference in means) among missing outcomes enough to induce clinically relevant bias in observed effect size;
   4. “As-treated” analysis done with substantial departure of the intervention received from that assigned at randomization;
   5. Potentially inappropriate application of single imputation.
3. Unclear risk. Insufficient information to permit judgment of ‘low risk’ or ‘high risk.’

**[rob_incomplete_text]**

Provide a description of the information used to code the risk of bias due to incomplete outcome data.

**[rob_report]**

**Selective Reporting:** What is the risk of bias due to ***selective outcome reporting***?

1. Low Risk of Bias. Any one of the following:
   1. The study protocol is available and all of the study’s pre-specified (primary and secondary) outcomes that are of interest in the review have been reported in the pre-specified way;
   2. The study protocol is not available but it is clear that the published reports include all expected outcomes, including those that were pre-specified (convincing text of this nature may be uncommon)
2. High Risk of Bias. Any one of the following:
   1. Not all of the study’s pre-specified primary outcomes have been reported;
   2. One or more primary outcomes is reported using measurements, analysis methods or subsets of the data (e.g., subscales) that were not pre-specified;
   3. One or more reported primary outcomes were not pre-specified (unless clear justification for their reporting is provided, such as an unexpected adverse effect);
   4. One or more outcomes of interest in the review are reported incompletely so that they cannot be entered into a meta-analysis;
   5. The study report fails to include results for a key outcome that would be expected to have been reported for such a study.
3. Unclear/Cannot Tell. Insufficient information to permit judgment of ‘low risk’ or ‘high risk.’

**[rob_report_text]**

Provide a description of the information used to code the risk of bias due to selective reporting.

**[rob_other]**

Other Bias: What is the risk of bias due to ***problems not covered elsewhere***?

1. Low risk. The study appears to be free of other sources of bias.
2. High risk. There is at least one important risk of bias. For example, the study:
   1. Had a potential source of bias related to the specific study designed used; or
   2. Has been claimed to be fraudulent; or
   3. Had some other problem.
3. Unclear/cannot tell. Insufficient information to assess whether an important risk of bias exists.

**[rob_other_text]**

Provide a description of the information used to code the risk of bias due to problems not covered elsewhere.

## Intervention and comparison groups

Create one record in this database for each of the intervention and/or comparison groups you selected earlier for coding. For example, studies with a single intervention group and a single comparison group will have two records in this section of the database.

##### GROUP IDENTIFICATION

**[groupid]**

Number each group consecutively within a study, starting with 1.

**[tvc]**

Select the type of group you are coding.

1. Intervention group
2. Control/comparison group

**[type]**

What type of services does this group receive?

1. Focal/primary bystander intervention program. There may be several focal programs in a study, as when two different types of programs are compared, both of which are expected to be effective.
2. Active treatment that is not a bystander program or IPV/sexual violence prevention program. This is a group that receives a sham treatment (e.g., watches a video on nature, receives nutrition information, diet intervention) intended to take the same duration as the focal intervention program, but does not involve any active bystander or IPV/sexual violence prevention/education components.
3. Inactive comparison condition. This is a group that receives no prevention program and gets only assessments.
4. Active business as usual. This is a group that receives “usual” active treatment (e.g., school-based sex education, college orientation that contains some sex education) that may be effective in preventing sexual violence but is not the focal treatment of the study. This treatment must be limited to services that the group would receive whether or not the research study was implemented (e.g., mandated college orientation). Programs that focus specifically on bystander behaviors or IPV/sexual assault are not eligible controls; however, sex education programs that may contain limited information on violence are eligible.
5. Other.

**[name]**

Program name. Write in the program name or label for this group. Please use the exact name the study authors report (many of the programs will be branded – Bringing in the Bystander, Mentors in Violence Prevention).

**[descrip]**

Program description. Write in a brief description of the treatment this group receives. As much as possible, quote or give a close paraphrase of the relevant descriptive text in the study report; always include page numbers from the report when appropriate. It is acceptable to copy and paste directly from the article as long as you include the information in quotations and provide a page number for the quotation.

##### Participant Characteristics

**[permale]**

Enter the percent of participants who identify as male in this group. Use -9 for cannot tell.

**[perfemale]**

Enter the percent of participants who identify as female in this group. Use -9 for cannot tell.

**[pertrans]**

Enter the percent of participants who identify as transgender in this group. Use -9 for cannot tell.

**[perwhite]**

Enter the percent of White participants in this group. Use -9 for cannot tell.

**[pernonwhite]**

Enter the percent of Non-White participants in this group. Use -9 for cannot tell.

**[perblack]**

Enter the percent of Black participants in this group. Use -9 for cannot tell.

**[perhisp]**

Enter the percent of Hispanic participants in this group. Use -9 for cannot tell.

**[perasian]**

Enter the percent of Asian participants in this group. Use -9 for cannot tell.

**[age]**

Enter the average age of the group using number of years. Use -9 for cannot tell.

**[agerange]**

Enter the age range of the group using “XX-XX” format. Use -9 for cannot tell.

**[ed_level]**

What is the grade/educational level for the majority of the group?

1. 7^th^-8^th^ grade
2. 9^th^-10^th^ grade
3. 11^th^-12^th^ grade
4. First year college
5. Second year college
6. Third year college
7. Fourth year college
8. Other/Cannot tell

**[perfrat]**

Enter the percent of participants in this group who reported being a member of a fraternity. Use -9 for cannot tell.

**[persor]**

Enter the percent of participants in this group who reported being a member of a sorority. Use -9 for cannot tell.

**[pergreek]**

Enter the percent of participants in this group who reported being a member of a fraternity or sorority. Note that this item is of value when the study authors do not break apart fraternity and sorority membership. Use -9 for cannot tell.

**[perathlete]**

Enter the percent of participants in this group who reported being a member of a school athletic team. Use -9 for cannot tell.

**[rape_myth]**

Enter the mean rape myth acceptance score for participants in this group at baseline. Use -9 for cannot tell.

**[empathy]**

Enter the mean victim empathy score for participants in this group at baseline. Use -9 for cannot tell.

**[per.sexual.victim]**

Enter the percent of participants in this group who reported being a victim of sexual assault (by any type of perpetrator). Use -9 for cannot tell.

**[per.physical.victim]**

Enter the percent of participants in this group who reported being a victim of physical assault perpetrated by a partner (i.e., hookup, date, relationship). Use -9 for cannot tell.

**[perknow]**

Enter the percent of participants in this group who report knowing someone who has been a victim of IPV and/or sexual assault. Use -9 for cannot tell.

**[efficacy_mean]**

Enter the mean bystander efficacy score for participants in this group at baseline. Use -9 for cannot tell.

**[perefficacy]**

Enter the percent of participants in this group who reported confidence in their ability to intervene when witnessing violence or its warning signs (measure at baseline). Use -9 for cannot tell.

**[intentions_mean]**

Enter the mean bystander intention score for participants in this group at baseline. Use -9 for cannot tell.

**[perintentions]**

Enter the percent of participants in this group who reported intentions to intervene when witnessing violence or its warning signs (as measured at baseline). Use -9 for cannot tell.

**[bystanderbx_mean]**

Enter the mean number of times participants in this group reported engaging in pro-social bystander behavior (i.e., intervening when witnessing any type of violence or its warning signs) at baseline. Use -9 for cannot tell.

**[perbystanderbx]**

Enter the percent of participants in this group who reported engaging in pros-social bystander behavior (i.e., intervening when witnessing any type of violence or its warning signs) at baseline. Use -9 for cannot tell.

##### Intervention group Characteristics

**[bystander_tx]**

To what extent did the intervention focus on bystander behavior?

1. Bystander intervention was the main focus of the program.
2. General sexual assault prevention program with some bystander content. Note that sexual assault programs should not include content on physical violence (these will likely constitute IPV prevention programs).
3. General IPV prevention program with some bystander content. Note that IPV prevention programs can focus on physical violence exclusively or on both physical and sexual violence. Programs focusing exclusively on sexual violence should be coded as sexual assault prevention programs.
4. Other/Cannot tell. Please bring these to the attention of the PI, as they may not be eligible.

**[mixedsex]**

What was the sex composition of the intervention group (in delivery setting)?

1. All females.
2. All males.
3. Single-Sex groups for mixed-sex sample.
4. Mixed sex.
5. Cannot tell.
6. NA – Individual Implementation.

**[sex_perp]**

How did the program conceptualize the sex of perpetrators of IPV/sexual assault?

1. Male perpetrators only.
2. Female perpetrators only.
3. Gender-neutral. Generic/ambiguous portrayal of sex or explicitly highlighting an equal possibility of male and female perpetrators.
4. Mostly male perpetrators.
5. Mostly female perpetrators.

**[sex_vic]**

How did the program conceptualize the sex of victims of IPV/sexual assault?

1. Male victims only.
2. Female victims only.
3. Gender-neutral. Generic/ambiguous portrayal of sex or explicitly highlighting an equal possibility of male and female victims.
4. Mostly male victims.
5. Mostly female victims.

**[gender_txt]**

Describe any intervention program content that addressed gender norms, rape myths, or other cultural norms as a root of IPV and/or sexual assault (or as an influence on bystander behavior). Where possible, please indicate whether gender/cultural norm content applies to sexual or physical violence. If a specific (branded) program is implemented (and a treatment manual or other program materials may be available) please clearly indicate this, as we may wish to obtain these materials for further coding.

**[format]**

In what format was the program typically delivered?

1. In-person. This involves some type of presentation or instruction delivered by one or more facilitators.
2. Video. This includes videos that are viewed on televisions or large screens as well as on computers (by groups or individual participants).
3. Web/computer delivered. This applies to non-interactive presentations that are viewed online as well as interactive computer-based programs. It excludes programs that primarily consist of participants viewing videos on computers (these should be coded as videos).
4. Ad/poster campaign. This applies to programs that are designed to promote awareness and contain minimal content (i.e., posters or flyers distributed across campus, brief emails sent to a listserv).
5. Other/cannot tell.

**[tx_context]**

In what context was the program typically delivered?

1. Individual with facilitator
2. Small groups (<10) with facilitator
3. Large group or whole classroom with facilitator
4. Individual alone (e.g., self-guided)
5. Other/cannot tell

**[facilitator]**

Who typically delivered the program?

1. Teachers or school administrators
2. Athletic coaches
3. Peer educators
4. Medical professionals (e.g., doctors, nurses, clinic staff)
5. Campus resource center staff (e.g., women’s center, sexual assault center, LGBTQ student center)
6. Non-campus community agency (e.g., domestic violence shelter staff/volunteers)
7. Mixed (no predominant type)
8. NA – Self-guided/implemented
9. Other/cannot tell

**[monitored]**

Monitoring of program implementation. Was the implementation of the program monitored by the author/researcher or program personnel to assess whether it was delivered as intended?

1. Yes, but no indication of feedback to facilitators. Do not infer that monitoring happened. Select “yes” ONLY if the report specifically indicates that implementation was monitored.
2. Yes, with indication that facilitators received feedback. Do not infer that feedback was provided. Select “yes” ONLY if the report specifically indicates that feedback was provided.
3. No indication that service delivery was monitored.

**[impprob]**

Implementation quality. Based on evidence or author acknowledgment, was there any uncontrolled variation or degradation in implementation or delivery of the program, e.g., high dropouts, erratic attendance, low compliance, program not delivered as intended, wide differences between settings or facilitators, etc. Note that this question has to do with variation in program delivery, not research contact. That is, there is no “dropout” if all participants complete treatment, even if some fail to complete the outcome measures.

1. Yes

2. Possible

3. No, apparently implemented as intended

**[impfid_txt]**

Implementation fidelity. Provide a description of any other implementation fidelity measures or assessments, including page numbers where appropriate.

**[frequency_contact]**

Approximate (or exact) frequency of contact between participant and facilitator or program activity. This refers only to the element of program that is different from what the control group receives.

1. Single-day program
2. Less than weekly
3. One to two times a week
4. Three to four times a week
5. Daily contact
6. Cannot tell

**[txwks]**

Duration of program in WEEKS. Approximate (or exact) mean number of weeks for the period over which participants received the program, from first to last intervention contact, excluding follow-ups designated as such. Divide days by 7; multiply months by 4.3; multiply years by 52; round to a whole number. Estimate for this item if necessary and if you can come up with a reasonable order of magnitude number (e.g., take the midpoint of a range if it is all that’s provided). Code -9 if cannot tell.

**[txhours]**

Duration of program as received in CONTACT HOURS. Approximate (or exact) mean number of contact hours for the period over which the participants received the program, from first program contact to last contact, excluding follow-ups designated as such. If dosage rates are provided multiply intended duration by dosage rate. Code -9 if cannot tell.

**[facilit]**

Facilitator training, preparation, or qualifications. Describe any information provided about the intervention facilitators’ training, level of preparation, or qualifications required for delivery. Provide page numbers.

**[incent]**

Incentives for recruitment or participation. Describe any incentives for participant recruitment and/or participation. Provide specific information about incentives (including dollar amounts), when available. Provide page numbers.

## Outcomes

**Step 1. Study and DV Identification**

Create one record for each dependent variable that you will be coding. For example, if the study measures bystander intentions and bystander behavior, you will have two dependent variable records. This is different from the number of times a dependent variable is measured in a study. For example, if the study measures bystander intentions before and after the intervention, you will have only one record here – for the bystander intention measure (but you will have two effect sizes for this outcome measure: one at pretest and one at posttest).

**[varid]**

Variable number. This number is an identification number for the dependent variable you are coding. Each dependent variable is numbered consecutively, within the study you are coding so that each has a unique VarNo for that study. If there is only one dependent measure for this study, you will create only one record in this worksheet, and the variable number will be 1. If there are three dependent measures, they will be numbered 1, 2, and 3.

**[dv.outcome.label]**

Enter a label for the variable you are coding. When possible, use the exact label that the study report authors use.

**[dvdes]**

Write in a brief description of the measure you are coding. This should include the authors’ label for this variable (e.g., rape myth acceptance, bystander efficacy, etc.), the instrument, the direction of scoring (e.g., lower scores are better), and information about what is being measured. Quote or closely paraphrase the description that is provided in the original report.

For group equivalence variables make sure the label describes successes in terms of bystander attitudes and behaviors (e.g., females, younger participants, participants in lower education/grade levels, participants who have been IPV/sexual assault victims, participants who know an IPV/sexual assault victim, participants with lower rape myth acceptance or greater victim empathy). When coding race always default to white v. non-white. Code non-white as a success.

**[dvmicro]**

What type of measure are you coding?

**Bystander Intervention Behavior**

1. Intervention – sexual violence – female victim
2. Intervention – sexual violence – male victim
3. Intervention – sexual violence – unknown victim sex
4. Intervention – physical violence – female victim
5. Intervention – physical violence – male victim
6. Intervention – physical violence – unknown victim sex
7. Intervention – unclear violence type – female victim
8. Intervention – unclear violence type – male victim
9. Intervention – unclear violence type – unknown victim sex

**Bystander Attitudes**

1. Bystander efficacy
2. Bystander intentions

**Violence Perpetration**

1. Sexual Assault Perpetration – female victim
2. Sexual Assault Perpetration – male victim
3. Sexual Assault Perpetration – unknown victim sex
4. Physical IPV Perpetration – female victim
5. Physical IPV Perpetration – male victim
6. Physical IPV Perpetration – unknown victim sex
7. IPV Perpetration – unclear violence type – female victim
8. IPV Perpetration – unclear violence type – male victim
9. IPV Perpetration – unclear violence type – unknown victim sex

**Violence Attitudes**

1. Victim empathy
2. Rape myth acceptance

**Victimization**

1. Sexual Assault Victimization
2. Physical IPV Victimization
3. Know a victim of IPV or sexual assault

**Other Predictor Variables**

1. Age
2. Sex
3. Race/ethnicity
4. Grade/education level
5. Other predictor of bystander behavior or IPV/sexual assault

**Prerequisite Skills for Intervening**

1. Noticing a sexual assault or its warning signs
2. Identifying a situation as appropriate for intervention
3. Taking responsibility for acting/intervening
4. Knowing strategies for helping/intervening

**[dvtype]**

Type of data collection used for outcome measure.

1. Pencil & paper questionnaire
2. Online/computer assisted questionnaire
3. In-person interview
4. Phone interview
5. Other
6. Cannot tell

**[dvdays]**

Number of Days: Enter the number of days over which outcome was counted. Enter -8 for lifetime measures (e.g., “ever” experienced sexual assault). Enter -9 if cannot tell. Multiply months by 30 (e.g., enter 3 months as 90 days).

**[icc]**

For cluster randomized trials, please enter the intraclass correlation coefficient (ICC) for each outcome variable coded. Code -8 for not applicable and -9 for cannot tell.

## Effect sizes

Although this is the final section of coding, it is a good idea to identify at least one codable effect size before you start coding a study, because studies that appear eligible frequently end up presenting data that cannot be coded into an effect size.

**[reportid]**

Report ID for this effect size. Indicate the report number (e.g., 22.02) for the report in which you found the information for this effect size. This is important so that we can find the source information for the effect sizes later on, if necessary, and is especially important for studies with multiple reports.

**[page]**

Page number for this effect size. Indicate the page number of the report identified above on which you found the effect size data. If you used data from two different pages, you can type in both, but use a comma or dash between the page numbers.

**[estype]**

Type of effect size you are coding.

1. Pretest and Posttest
2. Group equivalence

There are 3 types of effect sizes that can be coded: pretest, posttest, and group equivalence (or baseline similarity) effect sizes. They are defined as follows:

- **Group equivalence effect size**. Group equivalence effect sizes are used to code the equivalence of two groups prior to treatment delivery on (a) sex, (b) age, (c) race/ethnicity, or (d) predictors of pro-social bystander behavior. When multiple racial/ethnic group compositions are reported please report only white/non-white proportions (if not available, select another racial/ethnic group). When available, always code sex, age, and race/ethnicity. When multiple other predictors are reported select the three deemed to be most relevant (NOTE that behaviors are more relevant than attitudes/intentions).
- **Pretest effect size**. This effect size measures the difference between an intervention and comparison group before the intervention (or at the beginning of the intervention) on the same variable used as an outcome measure. Note: Use pretest data for different analytic samples if available. (e.g., separate pretest data for different follow-up waves).
- **Posttest/follow-up effect size.** This effect size measures the difference between two groups after intervention receipt on some outcome variable. Some posttests can occur during the intervention (after intake), immediately after the intervention ends, or any subsequent follow-up period after the intervention ends.

***Group Selection***

**[esgroup1] [esgroup2]**

Select the groups being compared in this effect size. Always select the focal prevention program to be ‘group 1.’

***Dependent Variable Selection***

**[varid]**

Select the dependent variable for this effect size.

**[estiming]**

Timing of measurement. Approximate (or exact) number of weeks **after the end of the intervention** when measurement occurred. Divide days by 7; multiply months by 4.3. Enter -9 if cannot tell, but try to make an estimate if possible.

***Effect Size Calculation and Data Entry***

It is now time to identify the data you will use to calculate the effect size and to calculate the effect size yourself if necessary.

You need to determine what effect size format you will use for each effect size calculation. There are two general formats you can use, each with its own section in FileMaker:

1. Compute ES from means, sds, variances, test statistics, etc.

2. Compute ES from frequencies, proportions, contingency tables, odds, odds ratios, etc.

Also note that within each of the above effect size formats, effect sizes can be calculated from a variety of statistical estimates; to determine which data you should use for effect size calculation, please refer to the following guidelines in order of preference:

1. Compute ES from regression coefficients with statistical controls for pretest measures and other potential confounding measures at baseline.
2. Compute ES from univariate descriptive statistics (means, sds, frequencies, proportions).
3. Compute ES from test statistics (t, F, Chi square).
4. If significance tests statistics are unavailable or unusable but p-values and degrees of freedom (df) are available, determine the corresponding value of the test statistic (e.g., t, chi-square) and compute ES as if that value had been reported. If you encounter these types of data, please see the PI.

Note that if the authors present both covariate adjusted and unadjusted means, you should use the covariate adjusted ones. If adjusted standard deviations are presented, however, they should not be used.

**[esfavor]**

Which group is favored?

For intervention-control comparisons, the intervention group is favored when it does “better” than the comparison group. The comparison group is favored when it does “better” than the intervention group.

For group equivalence comparisons, the intervention group is favored when it has a higher probability of pro-social bystander attitudes/behaviors than the comparison group (e.g., females, younger participants, participants in lower education/grade levels, participants who have been IPV/sexual assault victims, participants who know an IPV/sexual assault victim, participants with lower rape myth acceptance or greater victim empathy). For the time being, code non-white as a success.

Remember that you cannot rely on simple numerical values to determine which group is favored. For example, a researcher might assess rape myth acceptance, and report this as a score. A lower score is better than a higher score, so in this case a lower number, rather than a higher one, indicates a more favorable outcome.

Sometimes it may be difficult to tell which group is better off because a study uses multi-item measures in which it is unclear whether a high score or a low score is more favorable. In these situations, a thorough reading of the text from the results and discussions sections usually can bring to light the direction of effect – e.g., the authors will often state verbally which group did better on the measure you are coding, even when it is not clear in the data table.

Note that if you cannot determine which group has done better, you will not be able to calculate a numeric effect size. (You will still be able to create an effect size record—just not a numeric effect size.)

Select the group that has done “better”:

1. Intervention
2. Comparison
3. Neither, exactly equal
4. Cannot tell

**[esdata]**

Effect size derived from what type of statistics?

1. N successful/unsuccessful (frequencies)
2. Proportion successful/unsuccessful (percentage successful or not)
3. Means and SDs; means and variances; means and standard errors
4. Independent t-test
5. Chi-square statistic (1 degree of freedom)
6. Effect sizes as reported directly in the study
7. Other statistical approximation

**[esadj]**

For this effect size, did you use adjusted data (e.g., covariate adjusted means) or unadjusted data? If both unadjusted and adjusted data are presented, you should use the adjusted data for the group means or mean difference, but use unadjusted standard deviations or variances. Adjusted data are most frequently presented as part of an analysis of covariance (ANCOVA). The covariate is often either the pretest or some personal characteristic such as socioeconomic status.

- 1. Unadjusted data
  2. Pretest adjusted data (or other baseline measure of an outcome variable construct)
  3. Data adjusted on some variable other than the pretest (e.g., socioeconomic status)
  4. Data adjusted on pretest plus some other variables

**[estxasn]**

Assigned N for the intervention group

**[exctasn]**

Assigned N for the comparison group

**[estxobn]**

Observed N for the intervention group

**[exctobn]**

Observed N for the comparison group

**[estxmean]**

Mean for intervention group

**[esctmean]**

Mean for comparison group

**[estxsd]**

Standard deviation for intervention group

**[esctsd]**

Standard deviation for comparison group

**[escella]**

N successful for intervention group

**[escellc]**

N successful for comparison group

**[escellb]**

N failed for intervention group

**[escelld]**

N failed for comparison group

**[esindt]**

Independent t-value

**[eschisq]**

χ^2^ (df=1)

**[esauth]**

Standardized mean difference (SMD) reported by authors

**[esor]**

Odds ratio (OR) reported by authors

**[eshdse]**

Standard error (for SMD or OR) reported by authors

***Final Effect Size Determination***

**[es_fmsmd]**

Effect size value- standardized mean difference

**[es_fmor]**

Effect size value- odds ratio

Remember that you cannot rely on simple numerical values to determine which group has done better. For intervention-control comparisons, a positive effect size should indicate that the intervention group did “better” on the outcome measure than the comparison group, while a negative effect size indicates that the comparison group did “better” than the intervention group, and a zero effect size means that the two groups are exactly equal on the measure.

You must make sure that the sign of the effect size matches the way we think about direction, such that the effect size is positive when the intervention group (or posttest) is better and negative when the comparison group (or pretest) is better.

Effect sizes can range anywhere from around –3 to +3. However, you will most commonly see effect sizes in the –1 to +1 range.

Note: If the authors report an effect size, include that in your coding and use it for the final effect size value if no other information is reported. However, if the authors also include enough information to calculate the effect size, always calculate your own and report it in addition to that reported in the study.

**[esprob]**

Any problems coding this effect size?
